# Microspines in tropical climbing plants: a small-scale fix for life in an obstacle course

**DOI:** 10.1093/jxb/erac205

**Published:** 2022-05-13

**Authors:** Romain Lehnebach, Cloé Paul-Victor, Elisa Courric, Nick P Rowe

**Affiliations:** AMAP, Univ. Montpellier, CNRS, CIRAD, INRA, IRD, Montpellier, France; CIRAD, UMR Ecologie des Forêts de Guyane, AgroParisTech, CIRAD, INRA, Université des Antilles, Université de Guyane, Kourou, French Guiana, France; AMAP, Univ. Montpellier, CNRS, CIRAD, INRA, IRD, Montpellier, France; AMAP, Univ. Montpellier, CNRS, CIRAD, INRA, IRD, Montpellier, France; AMAP, Univ. Montpellier, CNRS, CIRAD, INRA, IRD, Montpellier, France; University of Cambridge, UK

**Keywords:** Attachment, climbing plants, GrowBots, microspines, robotics, searcher stems, sliding force, static force

## Abstract

Many climbing plants have microspines on their stems, which facilitate attachment and prevent slipping and falling from host plant supports. Extending via growth through complex environments and anchoring stems to substrates with minimal contact forces are key benefits for climbing plants. Microspines are also highly desirable features for new technologies and applications in soft robotics. Using a novel sled-like device, we investigated static and sliding attachment forces generated by stems in 10 species of tropical climber from French Guiana differing in size and climbing habit. Eight species showed higher static and sliding forces when their stems were pulled in the basal direction against a standard surface than in the apical direction. This anisotropic behaviour suggests that tropical climbers have evolved different ratchet-like mechanisms that allow easy sliding forwards but are resistant to slipping downwards. The presence of a downwards ‘stick-and-slip’ phenomenon, where static attachment is not significantly stronger than maximal sliding attachment, was present in most species apart from three showing relatively weak attachment by microspines. This indicates that diverse microspine attachment strategies exist in climbing plants. This diversity of functional properties offers a range of potential design specifications for climbing strategies on different substrates for artificial climbing artefacts.

## Teaser

Microspines are found on the stems of plants with different climbing strategies, particularly those that twine loosely and show a scrambling growth on irregular supports and in highly unstructured habitats.

## Introduction

Climbing mechanisms and attachment structures of plants have fascinated biologists for well over a century Darwin (1867). They are increasingly viewed as important functional traits in ecological studies (Putz, 1984, 1995; Putz and Chai, 1987; Hegarty, 1991, Gallagher and Leishman, 2012) and are also under scrutiny as sources for new bio-inspired technologies such as reliable attachment devices and adaptive fasteners in soft robotics. Attachment mechanisms and stem biomechanics of diverse tropical climbers should ideally be measured under natural conditions and soon after collecting samples that are still living. This often requires undertaking measurements under field conditions or in close proximity to the plants. In this paper, we integrate some of the conceptual and practical issues necessary for measuring the microspine attachment mechanisms of climbing plants under field conditions.

Climbing plants show a great diversity of attachment mechanisms by which they anchor themselves to surrounding supports. Twining of the leading stem and branches, attachment by tendril-like organs via twining or sticky pads, attachment by specialized roots, and a variety of hook structures are just a few of the diverse kinds of attachment (Isnard and Silk, 2009; Rowe and Speck, 2015). These broad categories have different functional advantages that can be specifically related to their individual mechanisms: stem twining and tendril twining attachments are effective for attaching to relatively slender objects up to certain diameters and do not work well on large-diameter supports (Putz, 1984; Putz and Chai, 1987; Hegarty, 1991; Hegarty and Caballé, 1991). Tendril climbers deploying adhesive pads (Steinbrecher *et al*., 2010, 2011; Seidelmann *et al.*, 2012) and root climbing mechanisms are very efficient at attaching to large-diameter stems and flat surfaces (Hegarty, 1991; Melzer *et al*., 2010, 2012). Some of these mechanisms depend on active growth processes and are probably less efficient for attaching to small twigs in the moving environment of the windswept tree canopy.

Hook-like attachment mechanisms include some of the simplest of plant climbing organs. They have also been the subject of interest as a distinct category of attachment mechanism. They vary from the millimetre to centimetre scale, such as in *Rosa arvensis* (Rosaceae) and *Asparagus falcatus* (Asparagaceae) (Gallenmüller *et al.*, 2015), climbing cacti (Soffiatti and Rowe, 2020), the hooks deployed by the acanthophylls (modified, whip-like, hook-bearing leaf rachises) and the flagella (modified, whip-like, hook-bearing fertile axes) of climbing palms (Isnard and Rowe, 2008a, b). Many hook-like climbing structures can be described as ‘passive’ climbing structures that do not undergo active tropisms or nastic movements compared with the ‘active’ responses of other attachment systems. They are effective at making attachments in wind-blown environments (Soffiatti and Rowe, 2020) and in situations involving some kind of movement, such as swaying, slipping, and falling of the climbing plant in relation to host supports. Many kinds of hook attachment rely on tensile forces to remain engaged, and in order for them to function as climbing aids there must be tension in the system for them to remain attached (Isnard and Rowe, 2008a; Rowe and Speck, 2015). In some cases, oscillating movements of the host stem and surrounding branches can unhook the attachment momentarily and reattach it at a position lower down the climbing stem on lower-placed hooks. This ratchet-like mechanism further tightens the attachment along the stem in tension. Arrangements of passive hooks deployed on whip-like stems and leaves of rattan palms are highly effective in anchoring via ratchet mechanisms of loosening, sliding, and tightening, resulting in a guy-rope-like system of secure attachment (Putz, 1990; Putz and Holbrook, 1991; Isnard and Rowe, 2008b, b).

Many hooks of climbing plants do not undergo movements, tropisms, or nastic movements like those of sensitive tendrillar organs. Instead, they rely on movements in the environment such as swaying or movement by wind to be deployed. The position and geometry of passive hooks on the deploying structure can be highly optimized for attachment in certain directions (Isnard and Rowe, 2008a; Rowe and Isnard, 2009). The mechanical architecture of the deploying structure itself in terms of rigidity, swaying, and the direction of stems oscillating in the wind likely play a role in optimizing ‘passive’ hooks for attachment in different environments (Rowe and Isnard, 2009). Some hook-like structures appear to share characteristics of both passive hooks and active tendrils, and are capable of thickening and consolidating attachment after interlocking with a support (Ewart, 1898; Putz and Holbrook, 1991; Rowe and Speck, 2015).

Hook-like attachment structures also exist as microspines or microhooks that are <1 mm in length and barely visible to the naked eye. Their arrangement on stems and leaves of climbing plants is known to enhance anchorage and fixation of the climber in the host vegetation. The temperate species *Galium aparine* (Rubiaceae) has served as an excellent model for demonstrating how plants can fine-tune microspine properties and their organization on the plant to facilitate anchorage or to limit sliding or falling in one direction but allow sliding against an object in another direction (Bauer *et al.*, 2011).

Hook-like microspines from plants have proved to be of great interest for bio-inspired applications as attachment and fastening mechanisms. Velcro is a widely used and successful example based on the hook-like spines of *Actinium lapper* (De Mestral, 1961), and such hook-like mechanisms are found in a wide variety of fruit dispersal mechanisms in plants (Gorb *et al.*, 2020). Studies on microspines of plants, as shown by the pioneering study of *G. aparine* (Bauer *et al.*, 2011), are not only providing insights into the biology of optimizing microspines for climbing but also providing models for bio-inspired technical innovations in robotics (Andrews and Badyal, 2014; Fiorello *et al*., 2018, 2020a, 2020b) as well as novel devices that attach to plant leaves for a variety of applications (Fiorello *et al.*, 2021). The deployment of microspines in plants appears to function well for a range of substrate properties and geometries, as well as in moving environments. Microspines can also provide instantaneous and reversible attachments that do not rely on ‘slow’ growth processes to function. Besides plants, microspines from animal biology are proving to be a huge source of bio-inspiration for technical applications from conventional climbing aids in robotics to rock-climbing applications in space exploration (Asbeck *et al.*, 2006; Liu *et al.*, 2015). The exact functional attributes of microspines and hook-like structures in both plants and animals is complex (Dai and Gorb, 2002; Gorb, 2008; Labonte and Federle, 2015).They depend on many factors, from the microstructure of the hooks themselves to their kind of deployment as well as the properties of the substrate. Furthermore, attachment can include one or more precise physical mechanisms, such as friction, dry adhesion, or interlocking with asperities on the substrate (Gorb, 2008). In this study using field-based measurements on a portable apparatus, we generally refer to the forces measured as attachment forces and attachment during friction rather than any more specific terms that would require observations at higher levels of precision. We also refer to both static and sliding forces generated during the experiments. We then discuss these forces with respect to putative life-history events and ecology involving different kinds of movement.

This study explores the attachment properties of searcher shoots of tropical rainforest climbing plants that bear microspines. Tropical vines and lianas are highly diverse and present a huge diversity of ecological strategies, climbing growth habits, climbing mechanisms, and attachment structures—far greater than that seen in temperate climbers (Gentry, 1991). Microspines are commonly observed on tropical liana shoots and their so-called ‘searchers’—the apical shoots of vines and lianas specialized for reaching and attaching to supports. They are particularly common in some tropical angiosperm families such as Dilleniaceae, which in Brazil are known locally as *cipó de fogo* (fire liana) because of the stem texture on young shoots, which produces burn-like marks if rubbed against the skin (Smith *et al.*, 2004). *Scleria secans*, known as razor grass, is a well-known albeit hurtful nuisance and a herbaceous monocotyledonous climber.

We investigate the attachment properties of young stems of 10 species of climbers bearing microspines. For convenience, we employ the term microspine rather than the term microhook. Microspine is a more inclusive term, which can include structures that are either hook-shaped, straight, or angled from their point of insertion on the plant stem. We use the term as a functional description rather than using one or more strictly botanical or morphological definitions such as thorns, spines, or prickles. Most of the structures observed in this work are probably most consistent with the definition prickle, since they are derived from the epidermis or just beneath it.

We explore a number of questions. The first of these is whether all climbing species bearing microspines show effective attachment and anti-sliding properties that are consistent with a climbing habit, and whether microspines also facilitate free sliding forwards during growth of the stem. Secondly, different climbing plants show wide differences in their capacity to reach across voids and spaces between supports, which is of great functional and ecological importance. We aimed to gain information on whether adhesive properties vary between species differing in size, mass, and length of searcher stem.

We argue that stem attachment is important for two levels of movement in climbing plants. We might refer to ‘slow’ and stop-start movements during the growth process, and ‘fast’ movements that are more linked with environmental processes such as wind action, branch swaying, slipping, and falling, which are ever-present in these environments. We investigate whether static attachment is always stronger than peaks of resistance during sliding or whether stems bearing microspines are capable of generating so-called ‘stick-and-slip’ mechanisms that create resistance during sliding, which is comparable to the forces generated during static attachment.

Further questions that we sought to answer are how microspine performance is linked to climbing modes such as twining and scrambling, and whether microspines increase climbing performance in highly unstructured environments. Finally, we summarize some of the findings and principles relevant for designing new bio-inspired attachment structures for soft robotics, in particular for new artefacts, known as GrowBots, which are capable of behaving like climbing plants (Gallentine *et al.*, 2020; Mazzolai *et al.*, 2020; Soffiatti and Rowe, 2020).

## Materials and methods

### Area studied

Samples of climbing plant searcher stems were collected from the lowland, tropical humid forest of northeast French Guiana on the Piste de St Elie, in the vicinity of the IRD (Institut de Recherche pour le Développement) research camp facility (5°30ʹN, 53°00ʹW) approximately 140 km northwest of the town of Cayenne.

### Choice of climbing species and determination of climbing habit

**Fig. 1.**
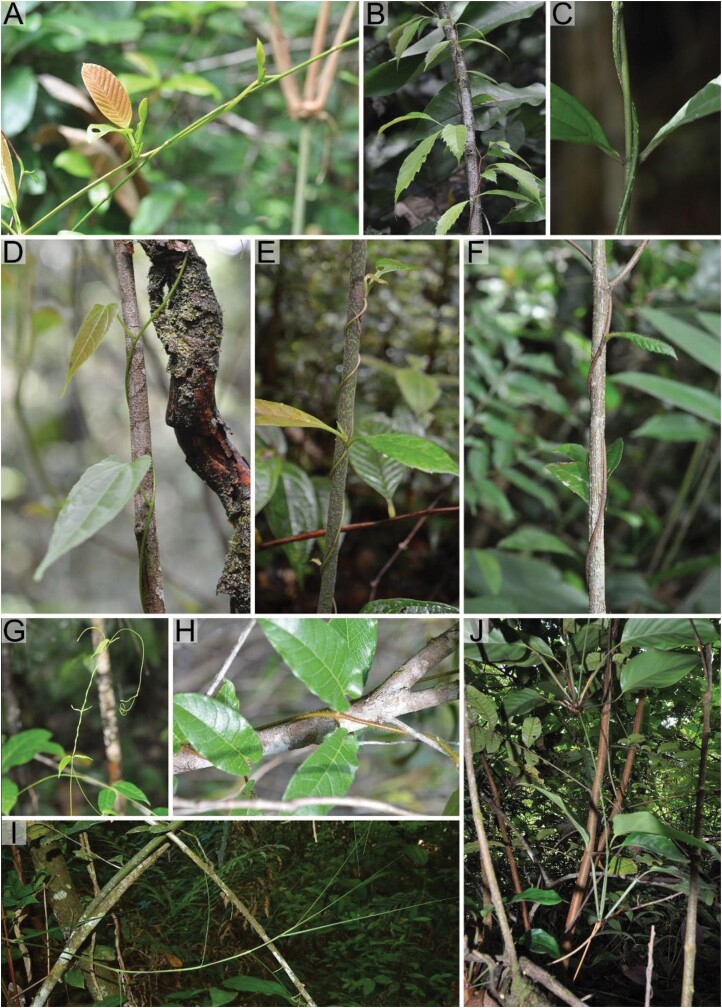
Overview of the climbing habits of the sampled species. Lax twining species [(A) *Davilla kunthii*, (B) *Doliocarpus dentatus*, (C) *Petrea volubilis*] wrap loosely around the supporting stems, whereas the stem of close twining species [(D) *Byttneria cordifolia*, (E) *Doliocarpus brevipedicellatus*, (F) *Davilla rugosa*, (G) *Mandevilla rugellosa*, (H) *Mandevilla hirsuta*] is almost always in close contact with the support as a result of closer and probably tighter twining. *Scleria secans* (I) and *Ischnosiphon centricifolius* (J) stems lean and arch over the surrounding vegetation and are considered to be scrambling species.

Searcher stems, defined as distal, self-supporting shoots of mature plants and of young, individual genets or ramets, were selected that showed the presence of microspines emerging from the surface of the stem and that showed a locking resistance when placed lightly on a finger (Supplementary Fig. S1) or when brushed against a leaf (Supplementary Video S1). Searcher stems were sampled for 10 species of climbing plants (Table 1) from forest gaps and forest margin habitats. For each species, we observed the exact kind of climbing mechanisms associated with the climbing habit (Table 1), such as twining versus scrambling along the surface of a supporting trunk, branch, twig, or leaves. If the behaviour was twining, we noted whether the climbing stems were twined closely (sometimes tightly) or wound loosely around host supports (Fig. 1; Supplementary Figs S2–S5).

**Table 1.** Stem characteristics of 10 climbing species that deploy microspines

**Species**	**N**	**Family**	**Climbing habit**	**Stem mass (g) [median (SD)]**	**Stem length (cm) [median (SD)]**	**Half stem mass**		**Stem diameter (mm)** ^a^	**Internode tested**
						**(g)**	**(mN)**		
*Byttneria cordifolia* Sagot.	9	Malvaceae	Close twiner	2.5 (15.0)	94.0 (12.0)	1.25	12.25	1.3 (0.28)	6
*Davilla kunthii* A. St.-Hil.	6	Dilleniaceae	Lax twiner	22.6 (12.5)	89.5 (23.7)	11.28	110.5	3.1 (0.98)	6
*Davilla rugosa* Poir.	11	Dilleniaceae	Close twiner	6.4 (14.6)	88.0 (13.1)	3.20	31.36	1.7 (0.48)	5
*Doliocarpus brevipedicellatus* Garcke	8	Dilleniaceae	Close twiner	8.7 (2.8)	87.5 (30.2)	4.35	42.63	1.9 (0.80)	5
*Doliocarpus dentatus* (Aubl.) Standl.	9	Dilleniaceae	Lax twiner	11.3 (12.7)	92.0 (19.0)	5.65	55.37	2.1 (0.57)	5
*Ischnosiphon centricifolius* L. Andersson	4	Marantaceae	Scrambler	10.1 (2.7)	164.5 (10.9)	5.01	49.12	2.7 (1.64)	2
*Mandevilla hirsuta* (Rich.) K. Schum.	7	Apocynaceae	Close twiner	6.0 (12.1)	83.0 (21.8)	3.00	29.4	1.7 (0.31)	4
*Mandevilla rugellosa* (Rich.) L. Allorge	5	Apocynaceae	Close twiner	2.0 (4.5)	49.0 (7.4)	1.00	9.8	0.9 (0.23)	5
*Petrea volubilis*L.	7	Verbenaceae	Lax twiner	5.0 (2.9)	91.0 (9.6)	2.50	24.5	1.9 (0.62)	3
*Scleria secans* (L.) Urb.	7	Cyperaceae	Scrambler	11.0 (2.0)	131.0 (11.8)	5.50	53.9	3.6 (0.34)	6

The climbing habit and the median of the fresh mass and length of the searcher stems are presented for each species. The stem diameter of the tested samples is also presented. The half stem fresh mass was used as an approximation of the normal force to set the weight of the friction sled. Samples for mechanical tests were selected from the same internode as numbered from the apex. This varied between species. N refers to the number of stems collected.

Stem diameter was measured at the part of the subapical linear part of the stem that deployed microspines and was sampled for friction tests. The number of samples used to compute the average of stem diameter correspond to N in Table 2.

### Morphology of searchers and approximation of normal forces for sliding tests for each species

**Fig. 2.**
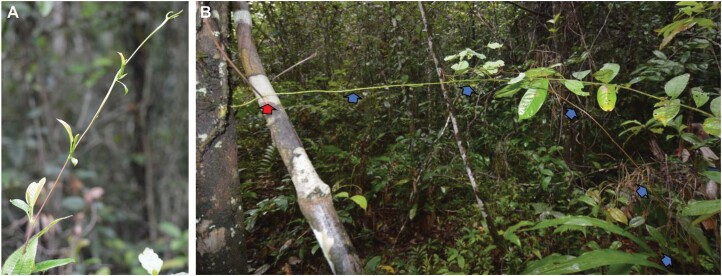
Liana searcher stems in the forest margin. (A) Typical self-supporting segment of ~35 cm of a liana searcher stem exhibiting long internodes and poorly developed leaves. (B) Searcher stem of a liana (blue arrows) reaching across from the parent plant (lower right) and spanning a void of 1.5 m until finding a support (red arrow).

All searcher stems selected were still in the self-supporting phase of development and not yet touching supports or attached (Fig. 2A). The fresh mass and length of the longest entire self-supporting searchers of each species were measured for each sampled stem (*n*=4–11) from the shoot apex to the base of the self-supporting segment. The latter was usually a branch point of the parent stem. Searcher shoots of different species differed in length, diameter, and mass (Table 1). We planned to apply a normal downward force to the test specimen against a standardized substrate during friction tests that would approximate the normal force of searchers for each species. The aim was to measure static and sliding forces that would be within the biological working range of the normal force that each species would apply to a support during its climbing life history (Fig. 2B). Friction tests would therefore compare the attachment performance between species that differed in terms of length, fresh mass, and diameter of the searcher stem. Each species was measured in friction tests with normal forces that were therefore relevant to their biological working range.

We approximated a resting force for each species that a searcher would exert on a support if it were supported at its base but resting against a support below its apex. Fig. 2B shows an example of a searcher that has spanned a void between the branch point with the parent plant and the support. The median force applied by an entire searcher stem in a fresh hydrated state was calculated for each species (*n*= 4–11). This force was divided by two to approximate the force created by a searcher that was attached to the parent plant at the base but rested on a potential support at its apex. This force was then used as the downward force applied to the plant stem during the static and sliding friction tests for each species.

This protocol was modified for assessing the resting force for searchers of *Ischnosiphon centricifolius* (Marantaceae) (Rowe *et al.*, 2006; Rowe and Speck, 2015). This species has a very different climbing growth habit compared with all of the other plants tested. The initially upright stems of this scandent cane-like monocotyledon are relatively long and very stiff, but after initial self-supporting growth the stems hinge at the flexible internodes. We approximated the resting force for a single distal stem internode as the median of the overall stem mass (*n*=4) divided by the number of cane-like internodes, resulting in an equivalent force of ~0.05 N.

### Sample selection for mechanical tests

**Fig. 3.**
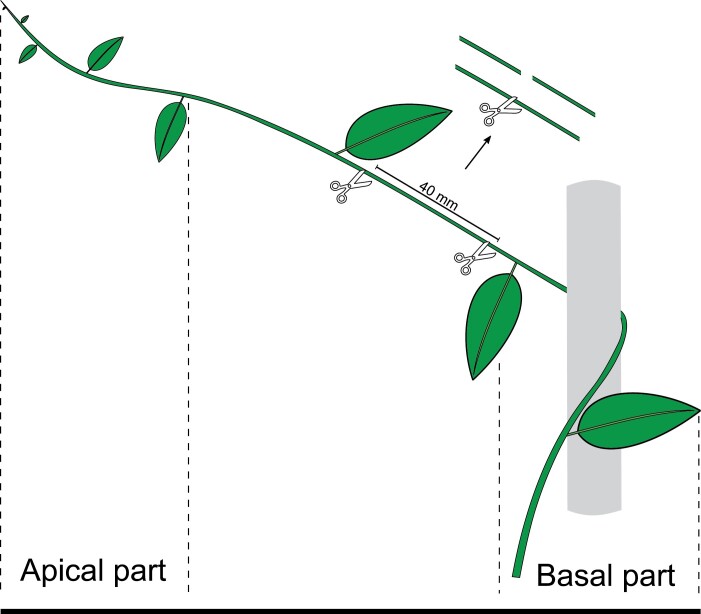
Position of the selected samples for mechanical tests of all species (apart from *I. centricifolius* ;see text). The apical part of the searcher stem consisting of soft tissues is too compliant and fragile to be tested with the sled friction test. The basal segment is generally too thick and quite often already wrapped around the support and has developed a periderm that has covered or replaced the microspine-bearing surface. Samples for mechanical tests were selected from the straightest part of the stem, beneath the apex and above the twined and more mature basal part. For each species, the same internode segment counted from the apex was selected, in order to standardize the developmental stage of the tested samples. The selected internode was cut into two equal parts that were used as sled ‘runners’ during the friction test (see Fig. 4).

Field observations suggested that microspine characteristics likely varied along the stem from the young green apex to the more mature basal part of the stem. The searcher stem base had often produced secondary tissues and in some species twined around a support. Selection of the tested stem segment, within a single internode, was standardized for each species, so that the developmental stage or age of the stem (in terms of internode number from the growing apex) was the same for each tested species (Fig. 3). A segment of stem ~40 mm in length was cut from a single internode of the microspine-bearing part of the stem. This segment was taken at a set number of internodes beneath the apex of the self-supporting stem (Table 1), depending on the species. This was often the straightest part of the searcher stem (Fig. 3). More apical parts of the stem in the growth zone often comprised soft green stem tissues and also already deployed hooks. These likely play a role in initial anchoring near the growth zone, but such young stages were too soft to easily fix to the sled-like support for friction tests (see below). The subapical part of the searcher stem bearing functional microspines was relatively mature and relatively stiff. Measurements were taken of the excised portions of this segment and the measured diameters were deemed to represent the functional stem diameter of the microspine-bearing portion of the searcher axis for each species.

The selected segment was cut into two equal parts (Fig. 3). For each species, friction tests were carried out on segments dissected from between 19 and 36 individual branch systems believed to represent branches from different individuals. Distinguishing between ramets and genets of lianas in natural forest conditions is particularly difficult. This is especially true in dense tangled habitats of forest margins where these species exist and proliferate. Many lianoid species show clonal life histories with highly indeterminate stem growth, resprouting, branching, and even physical detachment of resprouted individuals as separate individuals across distances and through different microhabitats of tens to hundreds of metres distance, or perhaps even more. This makes field sampling of unequivocally separate individuals very problematic. Searcher stems were accordingly sampled at different locations but similar habitats in terms of light exposure along the forest margin to minimize the likelihood of multi-sampling of clonal stems.

**Fig. 4.**
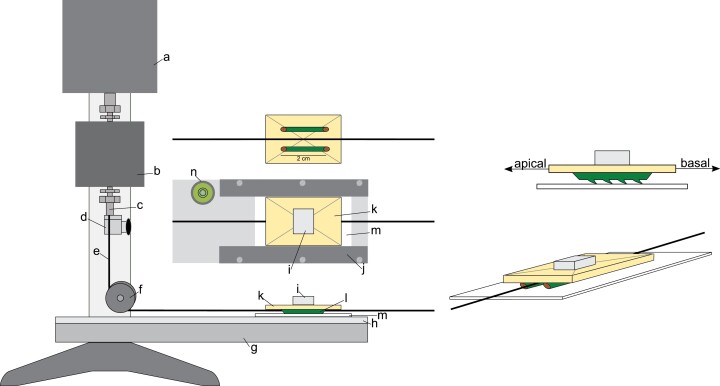
Experimental set-up for friction tests of plant stems. A portable universal testing machine (In-Spec 2200, Instron Corporation, Norwood, MA, USA) was equipped with an aluminium adjustable stand supporting a completely flat Perspex platform. A roughened (sanded; see text) sheet of Bristol card is held flat by plastic strips bolted down firmly along the edges of the Perspex platform. The pulling force is transduced horizontally to a balsa wood sled by a Kevlar thread via a pulley. Two stem samples with diagonally cut ends are glued in parallel on the lower side of the sled. The Kevlar thread is glued along the median line of the sled in a slight groove. The position of the Kevlar thread allows friction tests in both directions by simply rotating the sled and attaching the other end of the thread to the clamp. For each test, a weight is placed on to the upper side of the sled such that the whole mass of the sled (samples, balsa wood, and weight) approximates half of the fresh mass of the searcher stem (see text). a, Housing and motor of the testing machine connected to the computer interface; b, 10 N force transducer; c, crosshead; d, clamp; e, Kevlar thread; f, pulley; g, adjustable stand; h, Perspex platform; i, sled mass adjustment weight; j, screwed plastic strips holding the substrate flat; k, balsa wood board; l; stem samples oriented longitudinally with diagonal cut ends; m, Bristol card; n, bubble level.

Static and dynamic friction tests were carried out using a portable universal testing device (In-Spec 2200, Instron Corporation, Norwood, MA, USA) modified for sliding friction tests on plant stems. The field-testing device was based on a standard friction sled test set up where a test piece is pulled across a reference surface from the crosshead of the motorized device by using a 1 mm diameter Kevlar fishing line (Dracho Kev, Astucit) and a pulley (Fig. 4).

### Preparation of the sled and samples

Initial tests indicated that all attempts to pull a single stem segment against a flat reference surface became too unstable during sliding tests. Irregular movements and changes in orientation did not at all reflect the kinds of movement of an extended stem length either growing or slipping forwards or slipping downwards. Any manipulation or guiding of the specimen significantly modified and compromised the actual forces acting between the stem surface and the substrate. To ensure stable movement, we adjusted the set-up to test parallel segments of stem arranged like the runners of a sledge (Fig. 4).

The two parts of the selected segment were carefully glued to be exactly parallel to each other and oriented in the same apical–basal direction with respect to the direction of the microspines on a flat, rectangular strip of balsa wood 30 × 20 × 2 mm in size. Both ends of each segment were cut diagonally prior to each test to minimize the likelihood of the edge of the stems catching on to the asperities of the substrate surface.

The most stable configuration for towing the balsa sled with the pair of stem segments in the apical and then the basal direction was found by gluing the middle part of a length of Kevlar thread into a shallow groove on the lower surface and central axis of the balsa sled, which acted like a stabilizing keel. In this way the Kevlar thread extended from each end of the sled and could be conveniently fixed to the crosshead and then pulled towards the stem apex and then towards the stem base by simply clamping first one and then the other end of the thread to the In-Spec device crosshead without having to remount and reposition the stem segments (Fig. 4). The free end of the thread was arranged so it was pulled freely across a smooth, flat surface; control tests indicated that any drag from the thread had no effect on the friction measurements.

Before each test, the Kevlar thread was passed below and around a small pulley mounted on the sliding platform (Fig. 4) and attached to the crosshead of the In-Spec device. Control tests indicated that the pulley moved freely and exerted no additional resistance in the forces we were measuring. The platform was checked and adjusted to be perfectly flat with a bubble level. Control tests indicated that any slope had a large effect on the static and sliding forces measured with the sled and runner set-up. Before each test, the entire mass of the balsa sled plus the two stem ‘runners’ plus the glue were made up to one half of the median searcher mass of the tested species by placing small weights on to the top of the central part of the balsa sled (Fig. 4).

### Choice and preparation of substrate

In order to explore the different attachment performances of different species bearing microspines, we elected to analyse the static and sliding forces of the 10 species on one single type of substrate. Initial sled tests on leaves, cylindrical twigs, and bark surfaces—the most common host supports—indicated that surface textures and especially larger-scale ­geometries, such as twig diameter, leaf venation, and bark macro-topography, provoked very large variations in attachment performance based on substrate heterogeneity. Our aim was to provide a comparison of different species on the same substrate rather than of different species on different substrates. Following this study, it would be of great interest to examine how species attach to more diverse and especially natural substrates such as stem surfaces and leaves.

All samples were tested on a flat stationer’s cardboard surface [‘Fiches Bristol’, Oxford, France, size A4 (210 × 297 mm), 210 gsm]. This was chosen as a convenient, bio-sourced material consisting of plant fibres in a random pattern presenting cell-sized asperities with a mean diameter of 12.39 µm (SD=4.96) (*n*=30). This provided a flat, ‘cellular’ proxy for plant twig and branch surfaces, which make up the majority of natural supports for the climbing plant species in this study.

Initial tests indicated that attachment measurements made using the sled were very sensitive to changes in the angle or slope of the test substrate, and that tested substrates had to be carefully prepared and kept flat against the Perspex base with parallel plastic strips that were bolted down firmly (Fig. 4). Experiments in field conditions consistent with the test species’ habitat in terms of temperature and humidity meant that the substrate materials had to be carefully prepared. The sheets of Bristol card were trimmed to squares of 50 × 50 mm. Commercially available Bristol card has a smooth surface when new; initial tests indicated that the card surface would become roughened after three or four tests by the action of the microspines and so affect the measured performance of the microspines because of changes in the substrate.

Each card surface was pre-conditioned and roughened uniformly. Grade 240 emery paper was glued to the flat surface of a 500 g brass weight, and this was slid backwards and forwards five times (10 passes in all) to ensure that a uniformly roughened card surface was presented to the sled tests. Initial tests indicated that after nine tests, microspines would tend to rub grooves in the card. Prepared cards were therefore replaced every six to nine tests. Prepared card substrates were kept flat in hermetically sealed containers with silica gel so that they remained dry, since warping and softening of the card occurred if it was left for long periods in the open. Card substrates were taken out of the silica gel container and left in ambient humid conditions for 20 min before a run of tests. Trimmed card sheets were marked with regard to the orientation of all sheets in the packet of A4 sheets of card so that any bias in fibre direction within the package was likely the same for all tests.

### Sled test operation: basal and apical directions

Sled tests were routinely carried out first in the apical direction (sliding the stem segments towards their apex) and then in the basal direction (sliding the stems towards their base) (Fig. 4). For the first test, the sled was carefully placed on the cardboard test surface without pressing down more than the weight of the sled itself. The crosshead of the In-Spec device was adjusted so that the Kevlar thread was reeled in, so that it was not quite fully taut against the sled and suspended 2–5 mm above and parallel to the platform surface. The test was then started by activating the crosshead, advancing the sled at a speed of 3 mm s^–1^ for 20 mm and at a rate of 50 measurements per second. The crosshead was then stopped, the sled was rotated 180°, and the second Kevlar thread was attached to the crosshead. The protocol was then repeated for the test in the basal direction. The speed of 3 mm s^–1^ was used for all species as it tended to produce the most stable motion.

In most cases, sliding the stems towards the base produced a much stronger resistance than sliding them towards the apex. Testing the more resistant direction first would likely cause changes to the spine organization that would influence the smoother test. This was later borne out by scanning electron microscopic observations of tested stem surfaces showing fractured and dislocated spines. Therefore, the apical-then-basal sequence was chosen since it was likely that testing the ‘smooth’ orientation first would not influence the spine organization for the second test in the more resistant direction. Prior to each friction test, the diameter of the centre of each tested segment was calculated as the mean of one measurement in the vertical and one measurement in the horizontal direction, measured with digital callipers to 0.001 mm. The stem mass and sled mass were measured and made up to approximately half of the median searcher fresh mass for each species, with the exception of *I. centricifolius* (see above). Data were observed and checked in the field with the Instron Series IX software and recorded to hard disk during each test. Further calculations and data handling and presentation were undertaken with R (R Development Core Team, 2020).

### Parameters and their functional significance

The following parameters were extracted from the sled tests for all species.

#### Static friction (apical)

**Fig. 5.**
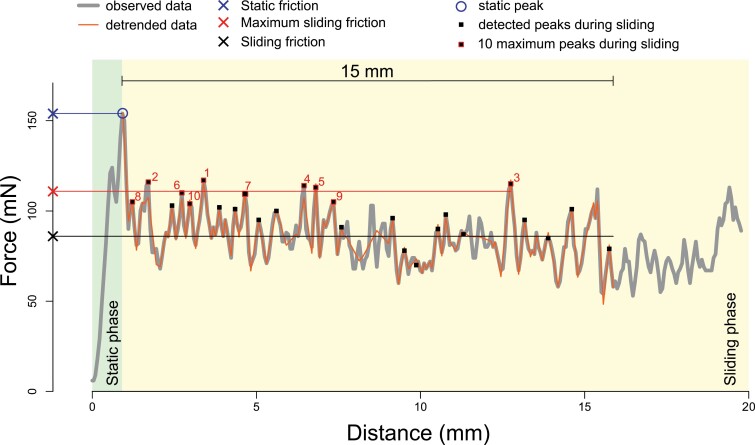
Representative force–distance curve obtained during a sled friction test illustrating the different parameters measured. The static phase (light green) corresponds to the increase of force until the force required to induce motion of the sled is reached (static force; blue cross). The sliding phase (light yellow) corresponds to the part of the curve where the sled actually moves on the surface. For each test, the computation of sliding parameters was performed with the data acquired along 15 mm, starting after the distance where the static peak occurred. Within this range, the mean of the force data was computed to estimate the average force during sliding (black cross). Within the 15 mm of the sliding phase, every positive and negative peak was detected by computing the difference between consecutive data points. The peak series was used for data detrending (orange line). The actual peaks (black squares) were detected using a peak identification algorithm. The number of detected peaks allowed computation of the peak frequency during sliding (N peaks per 15 mm). The average maximum force during sliding (red cross) was computed as the mean of the 10 highest peaks during sliding (red-framed black squares).

This is the force needed to induce motion of the sample in an apical direction (sliding the stem towards its apex). It was measured as the ­maximum value (in mN) of the first peak of the force–distance curve (Fig. 5). This parameter quantifies the resistance of the stem against its initial forward movement. Apical growth and elongation of the stem, as well as environmental factors (branch movements), enable ‘exploration’ of the climbing plant apex through complex networks of potential supports. Because of the time frame associated with the growth process (mm d^–1^ rather than mm s^–1^), apical static friction forces are relevant for assessing how easily a climbing plant can lengthen and explore forwards while it is in contact with a substrate without being impeded by its microspine system of attachment. It is also relevant to how easily a climbing plant stem can initiate a ratcheting movement forwards, resulting from the physical movement of branches (Putz, 1990; Isnard and Rowe, 2008a).

#### Static friction (basal)

This is the force needed to induce motion of the sample in a basal direction (sliding the stem towards its base). It was measured as the maximum value (in mN) from the first peak of the force–distance curve (Fig. 5). It quantifies the resistance of the stem surface against its initial basal movement. For stems climbing upwards, it is important for ensuring a locking mechanism on a vertical support and to prevent the onset of sliding (Supplementary Figs S1, S6).

#### Sliding friction (apical)

This is the force needed to maintain the sample in motion sliding towards the apex following initial movement from a stationary position. This was measured as the mean force (in mN) in the force–distance curve for a length of 15 mm (Fig. 5). It quantifies the ease by which the plant stem will move or slide forwards when in contact with a substrate. As noted above, forward sliding is probably more relevant to sliding and ratcheting movements caused by environmental movements, such as wind, rain, or swaying.

#### Sliding friction (basal)

This is the force needed to maintain the sample in motion sliding towards the stem base following initial movement from a stationary position. It was measured as the mean force (in mN) in the force–distance curve for a length of 15 mm (Fig. 5). It quantifies how easily the plant stem will keep sliding against a substrate when motion has started. This is of importance both to twining, climbing, and roving kinds of behaviour in general when the substrate moves in relation to the climbing plant stem (e.g. leaves and stems moving in the wind, which is often the case) and in cases when climbing plants start slipping from their support (Supplementary Fig. S6). Slipping downwards and falling off supports is a common and critical aspect of climbing. It is also a common occurrence during tree falls and branch falls and the physical perturbation in the vegetation surrounding these events. Around a tree-fall gap there is often much evidence of climbing stems that have been pulled off their supports by large-scale physical movements.

#### Static friction anisotropy

This derived parameter was calculated as the quotient of the static basal force and the static apical force. The value indicates how much more easily the plant can start a forward movement compared with a backward movement on a given support. It is a possible indication of how specialized the microspine adaptation is to the exact climbing mechanism of the plant. Climbing plants showing low anisotropies possibly have different climbing behaviours and rely on different attachment mechanisms than plants showing strongly anisotropic static movements.

#### Sliding friction anisotropy

This derived parameter was calculated as the quotient of the sliding basal force and the sliding apical force. Like the static force equivalent, it likely gives a means of comparing how plants with different climbing life histories differ in terms of how they slide forwards and backwards under external environmental movements. High anisotropies mean that stems tend to ‘stick’ when they are already sliding backwards, and low anisotropies would tend to indicate that stems tend to slide freely in both directions.

#### Maximum sliding friction

This parameter was calculated for the apical and basal directions during the sliding phase of the test. It provides a measure of the highest attachment forces (in mN) the plant surface is capable of during the sliding movement. This parameter is of interest to investigate whether different species can generate forces that approach the static force during sliding; in other words, it gives an indication of the ‘stopping’ potential of a plant stem to stop sliding in an apical or basal direction.

#### Peak frequency

This parameter was calculated for the apical and basal directions as the mean number of peaks (expressed as peaks mm^–1^) during the sliding part of the test. The measure gives an indication of the potential number of anchorage ‘events’ during the sliding, independent of the strength of the anchorage events. A higher peak indicates a microspine that has formed a momentary anchorage but has slipped or broken, whereas a lower peak indicates a ‘weaker’ or transitory anchorage that has slipped more easily because of the exact geometry or an easier failure of the hook.

### Statistical analysis

Statistical analysis was performed with R statistical software (R Development Core Team, 2020). Significant differences across species for each parameter of interest were assessed using parametric one-way ANOVA. When homoscedasticity and normality assumptions were not met, we used non-parametric Kruskal–Wallis rank tests. In cases of overall significant differences, multiple comparisons between species were performed by using post hoc Tukey’s honestly significant difference (HSD) tests with the ‘agricolae’ package (de Mendiburu, 2020). Within-species comparisons between two parameters measured according to both test directions [e.g. static force (basal) versus static force (apical)] were performed via comparisons of means. As the parameters of interest were measured on the same samples and the distribution of differences between parameters deviated from normality, we used one-sample paired Wilcoxon rank sum tests to assess significant differences between test directions for each species. We also used linear regression to assess stem mass and diameter as predictors of the static force in the basal direction. Finally, we fitted major axis regression to assess the slope of the relationship between the maximal force and the average force during sliding in both the basal and the apical direction using the ‘smatr’ package (Warton *et al.*, 2012).

## Results

### Overall climbing habit

The species studied encompassed a wide variety of different climbing behaviours. Most are twiners (Table 1; Fig. 1), which differed in the degree of dependence and closeness between the climbing stem and the supporting stem. Stems of lax twiners wrap loosely around the supporting structure, resulting in a few contact points between the climbing plant and the support (Fig. 1A–C; Supplementary Figs S4, S5). Close twiners, by comparison, twine more firmly around the supporting plant and are in contact with it along a lot of their length (Fig. 1D–H; Supplementary Figs S2, S3). The youngest apical portions of twiners are usually very delicate (they are flexible, turgescent, and deform easily in bending) with small diameters (stem diameter 0.9–3 mm) (Supplementary Fig. S7). Two species do not twine at all: the cane-like *I. centricifolius* and the razor grass *S. secans* have a more scrambling kind of growth form (Fig. 1I, J). *Ischnosiphon centricifolius* scrambles in understorey vegetation and also attaches to supporting branches by ‘hinge-like’ nodes, and has been described as a ‘branch-angle’ climber (Rowe *et al.*, 2006; Rowe and Speck, 2015). The two scrambling species also exhibit the widest-diameter stems of searchers of ~3 mm.

### Static and sliding forces

**Fig. 6.**
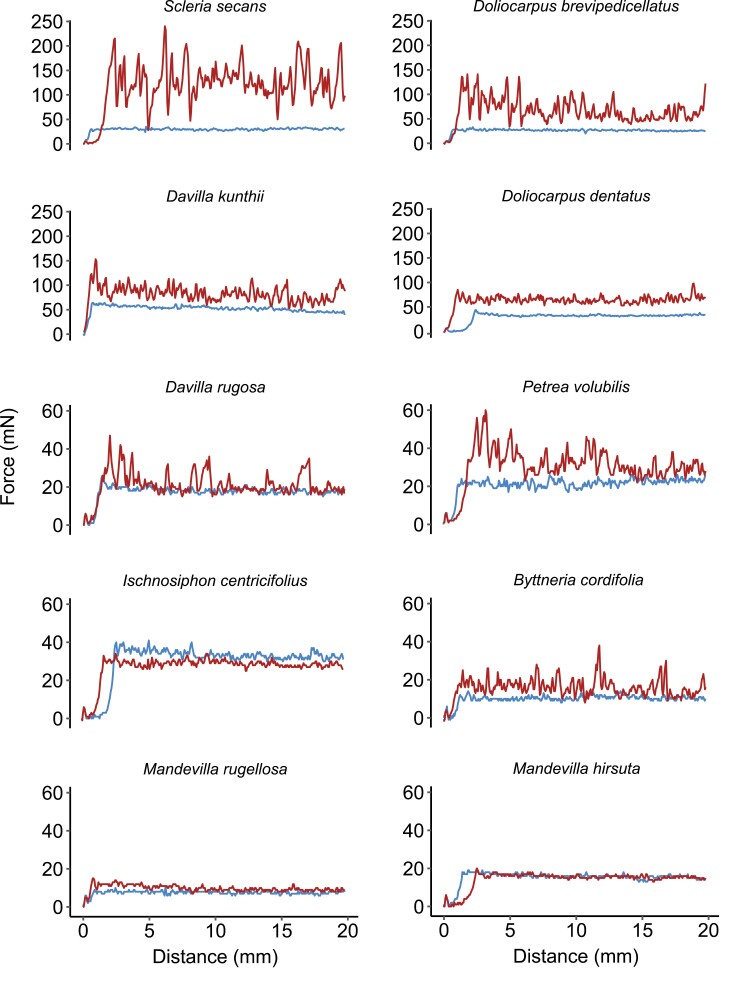
Typical species force–distance curves obtained during a friction test. The red and blue curves indicate forces in the basal and the apical direction, respectively. For convenience, the species have been separated into two groups differing in the magnitude of the measured force.

**Fig. 7.**
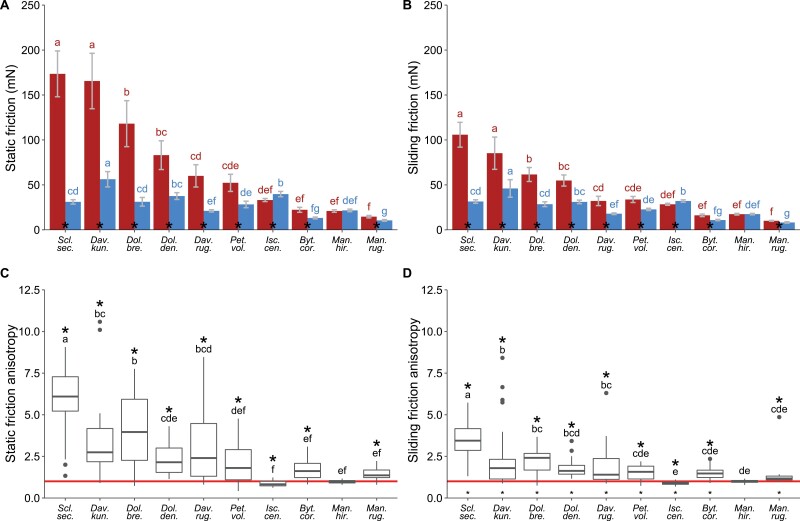
Between-species variation of static and sliding friction forces and their directional anisotropy. (A) Mean static friction forces and 95% confidence intervals observed along the basal (red) and the apical (blue) direction. The asterisks indicate significant differences (*P*<0.05) between the basal and apical directions assessed by paired Wilcoxon tests. Different blue and red letters indicate species that differ significantly (*P*<0.05) for the apical and basal directions, respectively, according to Tukey’s HSD test. (B) Mean sliding friction forces and 95% confidence intervals observed along the basal (red) and apical (blue) directions. Letters and symbols are as in (A). (C) Variations of static friction force anisotropy (basal/apical). Asterisks indicate species for which the anisotropy is significantly different (*P*<0.05) from 1, indicated by the red line). Different letters indicate species that differ significantly (*P*<0.05) according to Tukey’s HSD test. (D) Variations of sliding friction force anisotropy (basal/apical). Letters and symbols are as in (D).

The static friction force (the force required to produce motion) in the apical direction varied (Kruskal–Wallis, df=9, χ^2^=204.68, *P*<0.001) from 10.5 mN in *Mandevilla rugellosa* to a maximal value of 56.3 mN observed in *Davilla kunthii* (Figs 6, 7A). In the basal direction, the static force varied (Kruskal–Wallis, df=9, χ^2^=201.26, *P*<0.001) from a similar minimal level of 14.6 mN (in *M. rugellosa*) to over an order of magnitude higher at 173.5 mN (in *S. secans*).

Most plants showed a significant difference in static friction force between the less resistant apical direction and the more resistant basal direction (paired Wilcoxon test; *P*<0.001) (see Supplementary Table S1 for a complete description of the Wilcoxon tests performed in this study). However, the close twiner *Mandevilla hirsuta* (Figs 6, 7A), showed a similarly low apical and basal resistance (*P*=0.36). Another exception was *I. centricifolius,* the jointed, cane-like climber, which showed an opposite trend, with the apical static friction being significantly (*P*<0.001) greater than that in the basal direction (Figs 6, 7A).

The sliding friction force (the mean force required to keep the sled in motion) in the apical direction (Fig. 7B) varied (Kruskal-Wallis, df=9, χ^2^=207.69, *P*<0.001) from 8.2 mN (*M. rugellosa*) to 46 mN (*D. kunthii*). The sliding friction force was much higher in the basal direction, varying (Kruskal-Wallis, df=9, χ^2^=192.75, *P*<0.001) from 10 mN (*M. rugellosa*) to 105.8 mN (*S. secans*). Between species, static and sliding friction forces were positively correlated for both basal and apical directions (ρ=0.92, *P*<0.001 and ρ=0.93, *P*<0.001, respectively). Overall, the static friction was 1.2 and 1.6 times higher than the sliding friction for the apical and basal directions, respectively.

### Static and sliding anisotropy

Most species showed a marked anisotropy between static and sliding forces measured in both the apical and basal directions (Fig. 7C, D). Out of the 10 species tested, 9 exhibited significant anisotropy (Wilcoxon test, *P*<0.001), which was mostly positive (ratio >1, i.e. forces for the basal direction were higher than forces for the apical direction). *Ischnosiphon centricifolius* was the only species exhibiting a negative anisotropy (ratio <1). While anisotropy was observed for both static and sliding forces, across all species the mean static force anisotropy was ~1.3 times higher than the mean sliding force anisotropy. *Mandevilla hirsuta* was the only species not showing significant anisotropy for both static and sliding forces (ratio=0.97, *P*=0.38; ratio=1.00, *P*=0.95, respectively); *M. rugellosa*, another close-twining species, showed very low anisotropy (ratio=1.44, *P*<0.001; ratio=1.38, *P*<0.001, respectively).

### Stem mass and static force

**Fig. 8.**
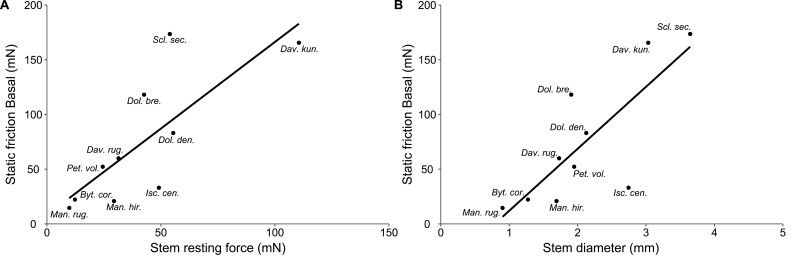
Static friction force in the basal direction and stem morphology. (A) Linear relationship between static friction force in the basal direction and stem resting force (*R*^2^=0.55, *P*=0.01). (B) Linear relationship between static friction force in the basal direction and stem diameter (R^2^=0.58, *P*=0.01).

Both stem mass and stem diameter were linearly related to the static friction force in the basal direction (*P*<0.01), but a wide range of static forces was observed for a given stem mass or diameter (Fig. 8A, B). Therefore, the predictions of the static friction force by the mass (*R*^2^=0.55, *P*<0.01) or the diameter (*R*^2^=0.58, *P*<0.01) are relatively limited. This suggests, perhaps not surprisingly, that the unexplained variance is most likely due to other functional differences between species and is likely influenced by other factors linked to attachment performance.

### Maximum sliding force

**Fig. 9.**
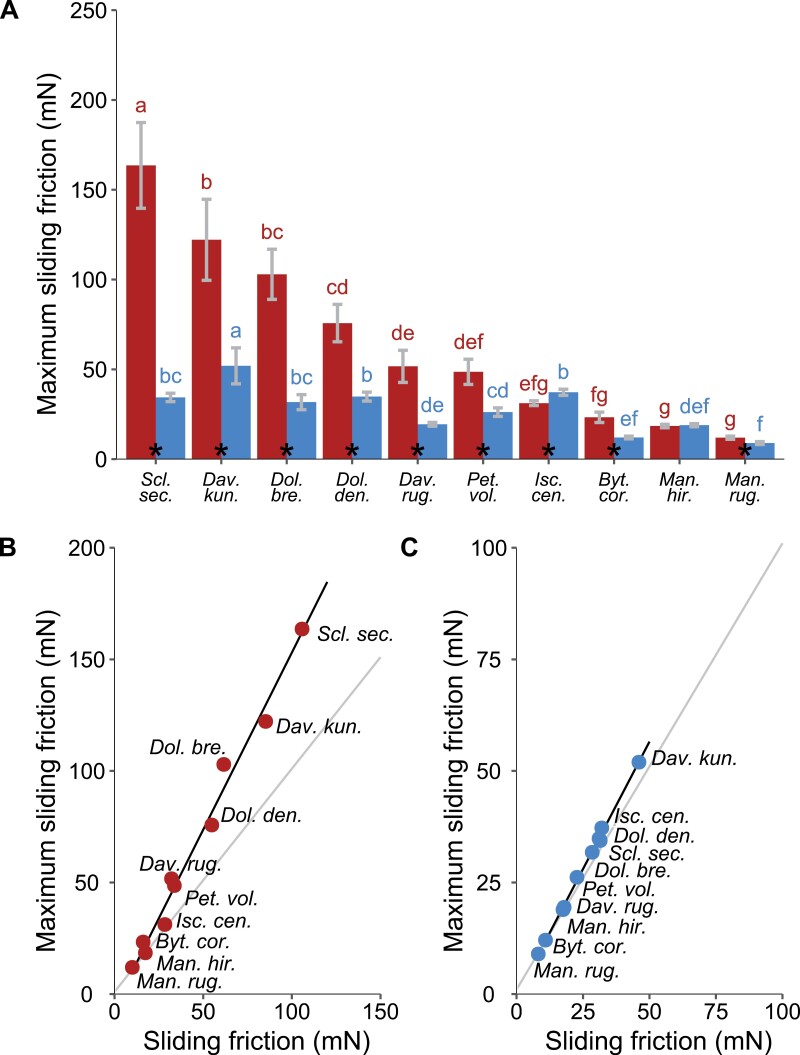
Between-species variation of friction forces during sliding. (A) Maximum friction force during sliding and 95% confidence intervals observed along the basal (red) and apical (blue) direction. Asterisks indicate significant differences (*P*<0.05) between basal and apical direction assessed by paired Wilcoxon tests. Different blue and red letters indicate species that differ significantly (*P*<0.05) for the apical and basal directions, respectively, according to Tukey’s HSD test. (B, C) Relationships between maximum friction force and average friction force during sliding in the basal (B) and apical (C) direction. The standard major axis (black line) fit and the 1:1 line (grey line) are shown. Regression parameters in (B): *y* =–5.65 + 1.59*x*, *R*^2^=0.98; regression parameters in (C): *y*=–0.55 + 1.14*x*, *R*^2^=0.99.

The maximum sliding friction force (mean of the 10 highest peaks) varied significantly between species (Fig. 9A) (Kruskal–Wallis, df=9, χ^2^=199.9, *P*<0.001 and df=9, χ^2^=208.96, *P*<0.001 for the apical and basal directions, respectively). Maximum sliding friction was linearly related to the average sliding friction in both the apical (*R*^2^=0.99, *P*<0.001) and basal (*R*^2^=0.98, *P*<0.001) directions (Fig. 9B, C). For the basal direction, the slope of the relationship was >1 [i.e. slope=1.59 (95% confidence interval 1.45; 1.73)], suggesting that across all species, the higher the average sliding friction force, the proportionally greater are the force peaks reached during sliding. In other words, the force–distance curves for basal directions show proportionally higher peaks when the average sliding friction force is high.

**Fig. 10.**
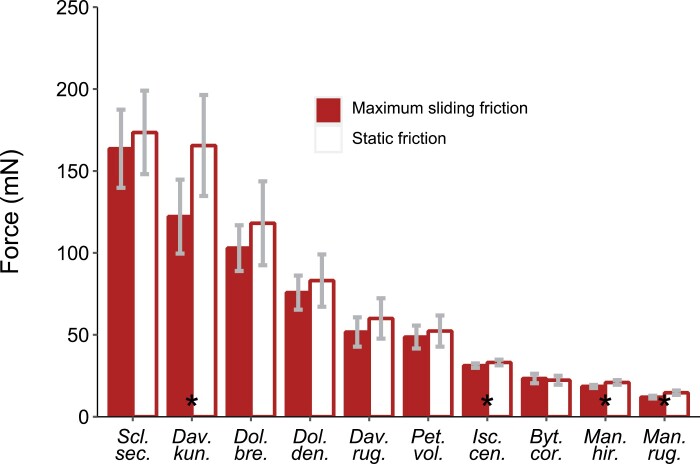
Comparison of static friction force and maximum friction force during sliding. The species-level mean value is presented with its 95% confidence interval. Asterisks indicate significant differences (*P*<0.05) between the static friction force and the maximum friction force during sliding assessed by paired Wilcoxon test.

This was not observed for the apical direction (Fig. 9C), where the slope of the relationship between the maximum sliding friction force and the average sliding friction force was close to 1 [i.e. slope=1.14 (1.10; 1.18)]. In other words, the force–distance curves for apical directions are more constrained (see Supplementary Fig. S8 for details of the friction tests for the apical direction). This is consistent with the slight variations of friction force observed during sliding in the apical direction compared with basal direction (coefficient of variation <15% for apical, up to 46% for basal). Out of 10 species tested, only four—*D. kunthii*, *I. centricifolius*, *M. hirsuta*, and *M. rugellosa*—showed a significantly lower maximum sliding friction force during basal sliding than their static friction force (Wilcoxon test, *P*<0.05). All the other species did not exhibit significant differences between their maximum sliding friction force and static friction force (*P*>0.14) (Fig. 10).

### Peak frequency

**Fig. 11.**
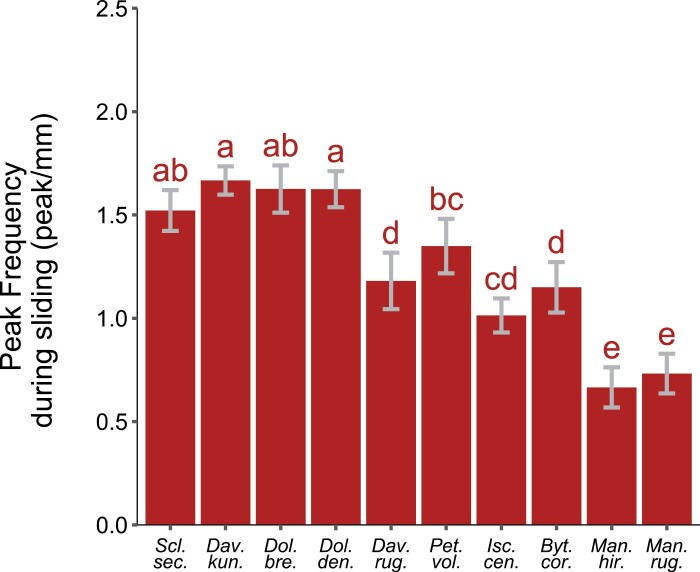
Peak frequency observed during sliding in the basal direction. The species-level mean value is presented with its confidence interval. Different letters indicate species that differ significantly (*P*<0.05) according to Tukey’s HSD test.

The peak frequency during sliding in the basal direction (Fig. 11) ranged from 0.7 peaks mm^–1^ in *M. hirsuta* to 1.67 peaks mm^–1^ in *D. kunthii.* Substantial variation between species was observed (ANOVA, df=9, *F*=40.031, *P*<0.001), but overall, the peak frequency did not differ significantly (>1.5 peaks mm^–1^) among species showing the highest static and sliding forces (*S. secans., D. kunthii, Doliocarpus brevipedicellatus*, and *Doliocarpus dentatus*) but was significantly higher in these species than in the other species, apart from *Petrea volubilis*.

### Spine morphology and orientation

**Fig. 12.**
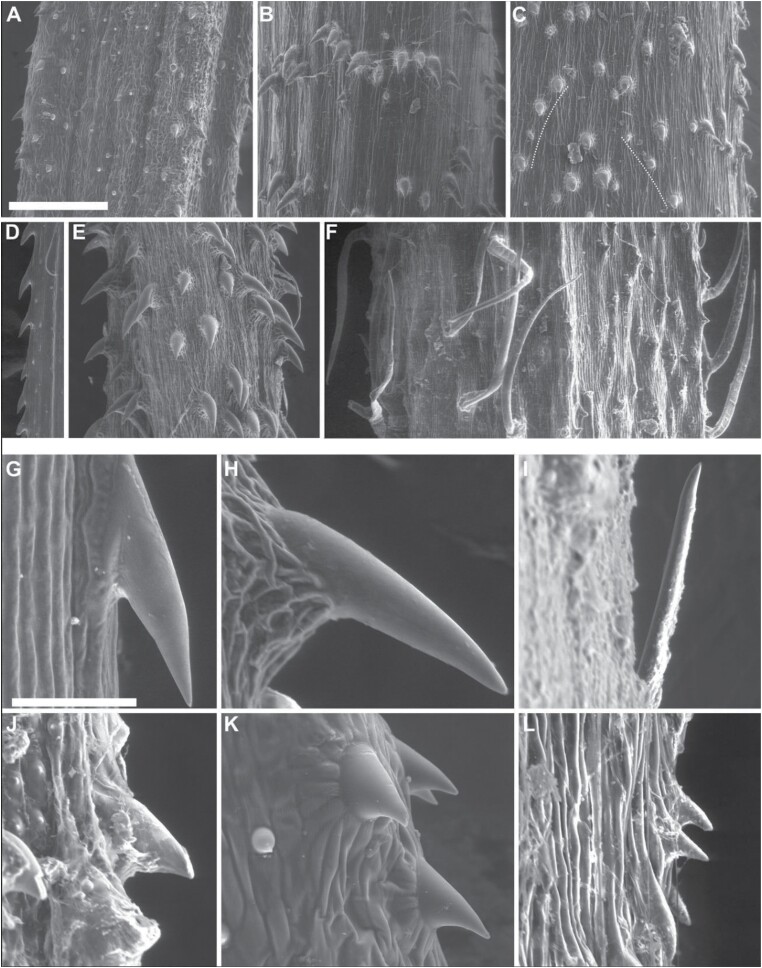
Overview of the microspine arrangement and morphological diversity of the sampled species. (A–F) Microspines are arranged in diverse ways, such as scattered on to the surface in *Doliocarpus dentatus* (A), clustered in *Doliocarpus brevipedicellatus* (B), in longitudinal files (indicated by the dashed lines) in *Davilla kunthii* (C), along the ridge of the stem in *Scleria secans* (D), in diagonal lines in *Davilla rugosa* (E), or interspersed with hairs in *Mandevilla hirsuta* (F). (G–L) Microspine morphological features, such as insertion angle, length, or tip sharpness varies between species. Most of the species exhibit microspines pointing basally [(G) *S. secans*, (H) *D. rugosa*, (J) *Petrea volubilis*, (K) *D. dentatus*, (L) *Mandevilla rugellosa*], except for *Ischnosiphon centricifolius* (I). Scale bar=500 µm (A–F) or 100 µm (G–L).

Microscopic observation revealed a variety of arrangements of microspines on the stem surface (Fig. 12). Microspines can be scattered on the surface without any marked pattern, as observed in *D. dentatus* (Fig. 12A); they can be arranged as clusters or in verticils, as in *D. brevipedicellatus* (Fig. 12B); in files more or less oriented along the stem axis, as in *D. kunthii* (Fig. 12C); along the ridges of the stem, as in *S. secans* (Fig. 12D); in diagonal lines, as in *D. rugosa* (Fig. 12E); or interspersed with hairs, as in *M. hirsuta* (Fig. 12F). On the stem surface, microspine density varied from 6 mm^–2^ (in *S. secans*) to 118 mm^–2^ (in *M. rugellosa*) (Supplementary Table S2). Overall, microspines were small (<1 mm), varying in length between 50 µm and 160 µm (Fig. 12G–L) and in diameter between 32 µm and 73 µm (Supplementary Table S2). Microspines pointed basally in all sampled species except for *I. centricifolius*, where they pointed apically (Fig. 12I). Microscopic details of all the species sampled are shown in Supplementary Fig. S9.

## Discussion

### Frictional anisotropy and growth habit

We identified a variety of different anisotropic properties in different climbing growth habits. Species that we identified in the field as lax twiners, which often twined loosely around supports (Fig. 1A–C; Supplementary Figs S4, S5), showed highly anisotropic attachment properties. This kind of loose twining or winding growth on irregular supports has been previously discussed (Rowe and Speck, 2015). The lax twining forms described here likely rely on microspines for initial attachment and to prevent slipping downwards before and after the stem has initiated its lax twining around the support. These findings are consistent with the idea that friction is important for stem twining (Putz and Holbrook, 1991; Silk and Holbrook, 2005; Goriely and Neukirch, 2006) and that some twining species have evolved structures on the stem surface to improve and optimize the friction of the stem (Silk, 1989; Silk and Holbrook, 2005; Isnard *et al.*, 2009).

Our field observations also suggest that some species have evolved a close and (although not measured in this study) ‘tight’ twining mechanism around the support (Fig. 1D–H; Supplementary Figs S2, S3). Observations in the field unequivocally suggest that such twining is closely wound and tight to the support. It is notable that the twining species with the lowest anisotropy and smaller static and sliding forces, such as *M. rugosa*, *M. hirsuta*, and *Byttneria cordifolia*, tended to be close twiners. The species of *Mandevilla* showed especially low anisotropy and low attachment forces, suggesting that these close twiners rely little on microspines for optimizing stem attachment.

Twining species differ in terms of frictional properties as well as closeness of twining to supports, and this diversity exists within the same areas of disturbed forest margin conditions in French Guiana that we studied. Laboratory measurements of twining stems (Silk, 1989; Silk and Holbrook, 2005; Isnard *et al.*, 2009) as well as mathematical models (Goriely and Neukirch, 2006) of twining behaviours underline the importance of tensile forces in the attachment of twining stems. Previous field workers have also remarked on the importance of the ‘availability of supports’ and ‘trellis availability’ for climbing plants to grow up to the light in tropical forests (Putz, 1984). Twining growth forms in tropical forests in Barro Colorado, Panama, have been described as showing preferences for ‘smooth boles of saplings and small trees’ (Putz and Holbrook, 1991). We suggest that lax twining with the aid of microspines potentially allows attachment to a wider range of available, heterogeneous supports. For example, some of our observations show evidence of loose or lax twining around irregularly branching, leafy stems (Supplementary Figs S10, S11), which would not accommodate the kinds of tensile or ‘squeezing’ forces of the climbing stem that have been shown in controlled laboratory experiments on smooth cylindrical supports (Scher *et al.*, 2001; Silk and Holbrook, 2005; Isnard *et al.*, 2009). This functional diversity within twining climbing growth forms is consistent with observations that success in reaching higher within a tropical forest depends on not only the availability of supports but also the ability to attach to different kinds of support (Putz, 1984).

The non-twining species *S. secans* (razor grass), which clambers irregularly on surrounding branches, showed highly anisotropic properties and by far the highest attachment forces on the card substrate. This suggests that in the absence of a close or lax twining behaviour, microspines are important for attachment. The climbing habit of *S. secans* could be referred to as ‘microhook climber’, which is efficient at attaching to a wide range of supports (Rowe *et al.*, 2006). Scrambling and hook-climbing species potentially have different support preferences from twining species (Putz, 1984, 1995; Putz and Chai, 1987; Hegarty, 1991; Putz and Holbrook, 1991; Rowe *et al.*, 2006; Rowe and Speck, 2015). Observers have emphasized the fact that scrambling and hook climbers depend more on small-diameter supports and cluttered, heterogeneous supports and trellises (Putz, 1984; Hegarty, 1991; Hegarty and Caballé, 1991; Putz and Holbrook, 1991). Our observations on frictional properties suggest that reliance on microspine attachment is possibly more prevalent in scrambling and lax-twining species than in close, tight-twining species.

**Fig. 13:**
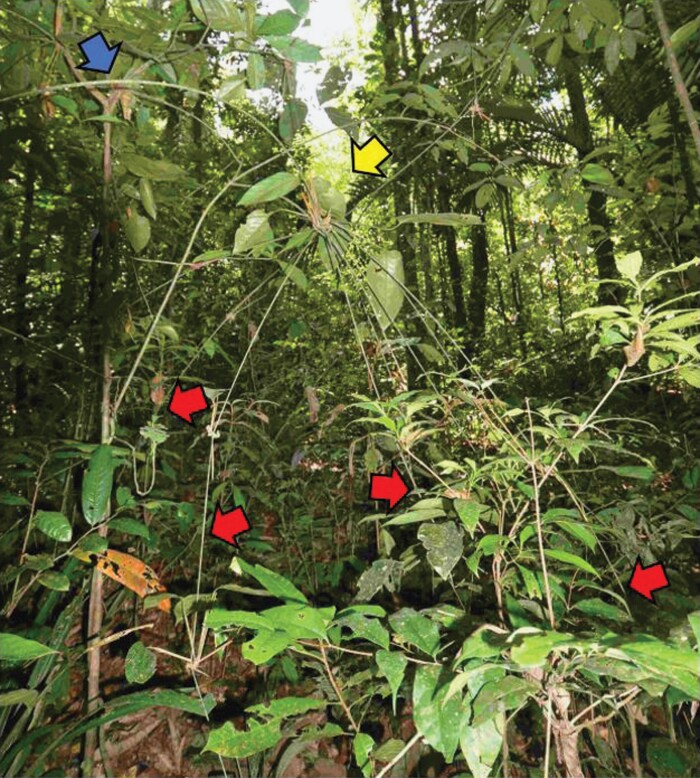
Growth habit of *Ischnosiphon centricifolius*. A long arching stem (blue arrow) extends across a void from a nearby tree support. At least eight branches are suspended from a node (yellow arrow), and ‘spider-like’ hanging stems are clinging to and held in place (red arrows) against the neighbouring leaves and twigs of understorey shrubs.

The cane-like branch-angle climber *I. centricifolius* showed opposite anisotropy, with microspines actually pointing apically on leading shoots. This organization generated somewhat higher forces in the apical direction compared with the basal direction. The climbing habit of *I. centricifolius* (Rowe *et al.*, 2006; Rowe and Speck, 2015) differs markedly from that of the other species tested. Climbing is effected by stiff, cane-like stems that initially grow upwards and then branch. It colonizes patches of understorey forest, spanning across large gaps, and inter-tangling with branches of the understorey plants via hinge-like flexible nodes. Apical shoots hang down against branches and leaves of the understorey (Fig. 13). Although further measurements would be necessary, apically orientated spines would likely position these distal shoots above host leaves rather than sliding or ratcheting themselves underneath them.

Dense, cluttered, and unstructured situations are typical of many tropical forest locations such as forest margins, tree-fall gaps, and storied forest canopy layers. Lax twining to scrambling mechanisms of climbing aided by microspine attachment are arguably well adapted to navigating highly unstructured and unpredictable supports in three-dimensional space (Supplementary Fig. S12). Close-twining species are arguably more adapted to attach to uniformly shaped supports. Branch points of the main host stem and randomly positioned branches, twigs, and leaves that are positioned against the main host stem (Supplementary Figs S10, S11)—a very common occurrence in these dense forest habitats—potentially interrupt the close twining mechanism and impair its ability to maintain a close contact with a support.

### Static and sliding forces: movement by growth, roving, bracing, and climbing

Static attachment forces via friction likely play a crucial role for maintaining attachment during slow, stop-start movements during plant growth (extension by growth of the axis) as well as slow movements (nastic, circumnutational, and oscillatory movements) of climbing stems just prior to complete gyres around the host support (Scher *et al.*, 2001; Silk and Holbrook, 2005; Isnard and Silk, 2009; Rowe and Speck, 2015). Laboratory studies (Filippov and Gorb, 2013) on individual microspines have shown that microspine stiffness and the rigidity and the basal connecting joints of microhooks play an important role in microspine properties. The more complex microspines of the hop plant (*Humulus lupulus*) that can pivot at the base via a lever-like mechanism probably optimize a ‘pinching’ kind of attachment during early contact with the host stem surface (Isnard and Silk, 2009). These mechanisms support the idea that the presence of microspines on the subapical, straight parts of leading shoots (the parts of the axis tested in this study) will initiate contact attachment before and during the completion of entire gyres in the twining process (Supplementary Fig. S7). Sled experiments were designed to apply a normal downwards force that approximated the ‘resting force’ for searcher shoots of each species at their maximum length or reach (Hattermann *et al.*, 2022). The results reported in this paper essentially represent the part of the stem that initiates initial contact to host supports and then twines around the host stem. It was not possible to carry out stable sled tests on already curved twining segments of stem, but the results nevertheless represent the frictional properties essentially deployed to initiate twining.

With the exception of *M. hirsuta* (a close twiner) and *I. centricifolius* (a monocotyledonous branch-angle climber), all the species we studied generated a basal static force exceeding half of the searcher mass. This meant that most searcher shoots generate sufficient frictional attachment to anchor themselves at maximal reach length. This feature could be viewed as a kind of safety factor whereby different species ensure attachment while spanning for their respective stem size and mass. A relationship between force of attachment and length of stem and the likelihood that that attachment will prevent falling is likely present in other kinds of climbing plant mechanisms. Pioneering studies by Ewart in the 19th century observed that different species developing much larger hook-like structures tended to show a link between the strength of hook attachment and the length of stem supported (Ewart, 1898; Isnard and Silk, 2009).

Initial frictional attachment with potential supports provided by light resting forces are advantageous for climbing plants. They act as starting points for climbing that develops additional attachments, such as twining (Scher *et al.*, 2001), petiole and branch-angle anchorage (Gallenmüller *et al.*, 2009), or root-climbing in certain cacti (Soffiatti and Rowe, 2020). They function in a time window during which stronger attachment mechanisms develop via slower growth processes. The mechanism is likely important for optimizing initial contact prior to forming a complete gyre around a support in twining species. These mechanisms are similar to so-called ‘bracing’ in technical applications, where technical artefacts such as robotic arms need to initially fix themselves to supports in order to navigate through unstructured environments (Book *et al.*, 1984; Walker, 2015).

### Sliding forces and movement

Sliding forces are likely crucial during fast movements when shoots, branches, and leaves oscillate in the wind, are pounded by heavy rain, or slip during tree falls and branch failures, as well as in falls and slipping of the climbing plant itself. All of these ‘faster-than-growth’ processes are constant ongoing phenomena for climbing plants in tropical rainforests. Most species showed greater anisotropy during static friction than during sliding friction (Fig. 7C, D; Table 2) except for *M. hirsuta*, *M. rugosa*, and *I. centricifolius*. Looking at the peak forces during sliding in more detail, however, indicated that nearly all species (other than the same three species listed above) showed little or no difference between the 10 maximal force peaks during the 15 mm sliding test and the static force peak.

**Table 2.** Summary of the parameters estimated during the friction tests

**Species**	***n***	**Static force (mN)**		Sliding force (mN)			Mean 10 peaks (mN)			**Peak frequency (peak mm** ^**–1**^)		**Stem diameter (mm)**
		**Apical**	**Basal**	**Anisotropy**	**Apical**	**Basal**	**Anisotropy**	**Apical**	**Basal**	**Apical**	**Basal**	
*Byttneria cordifolia*	26	13.2 (12.2; 14.2)^fg^	22.3 (19.5; 25)^ef^	1.7 (1.5; 2)^ef^	10.9 (10.2; 11.6)^fg^	16.1 (14.7; 17.5)^ef^	1.5 (1.4; 1.7)^cde^	12.1 (11.3; 12.8)^ef^	23.3 (20.4; 26.2)^fg^	0.7 (0.6; 0.7)^d^	1.1 (1; 1.3)^d^	1.3 (0.28)
*Davilla kunthii*	28	56.3 (47.7; 64.8)^a^	165.5 (134.7; 196.4)^a^	3.4 (2.5; 4.3)^bc^	46.0 (36.3; 55.8)^a^	85.2 (67.3; 103.2)^a^	2.5 (1.7; 3.2)^b^	52 (41.9; 62)^a^	122.2 (99.6; 144.7)^b^	1.3 (1.2; 1.3)^a^	1.7 (1.6; 1.7)^a^	3.1 (0.98)
*Davilla rugosa*	36	21.1 (19.8; 22.4)^ef^	60 (47.7; 72.3)^cd^	3.1 (2.3; 3.8)^bcd^	17.9 (36.3; 55.8)^a^	32.1 (26.9; 37.3)^cd^	1.9 (1.5; 2.2)^bc^	19.4 (18.4; 20.5)^de^	51.7 (42.7; 60.6)^de^	0.7 (0.7; 0.8)^d^	1.2 (1; 1.3)^d^	1.7 (0.48)
*Doliocarpus brevipedicellatus*	23	31.2 (26.3; 36)^cd^	118.1 (92.5; 143.7)^b^	4.1 (3.1; 5)^b^	28.5 (25.8; 31.2)^cd^	61.6 (53.8; 69.3)^b^	2.2 (1.9; 2.6)^bc^	31.8 (27.6; 36)^bc^	102.9 (88.9; 116.9)^bc^	1 (0.9; 1.1)^ab^	1.6 (1.5; 1.7)^ab^	1.9 (0.80)
*Doliocarpus dentatus*	25	37.6 (33.8; 41.3)^bc^	83.1 (67.1; 99.1)^bc^	2.2 (1.9; 2.6)^cde^	31.0 (29.1; 33)^bc^	54.8 (48.7; 61)^bc^	1.8 (1.6; 2)^bcd^	34.8 (32.3; 37.3)^b^	75.7 (65.3; 86.1)^cd^	1.1 (1; 1.2)^a^	1.6 (1.5; 1.7)^a^	2.1 (0.57)
*Ischnosiphon centricifolius*	33	39.8 (36.8; 42.7)^b^	33.1 (31.4; 34.8)^def^	0.9 (0.8; 0.9)^f^	32.1 (30.7; 33.4)^b^	28.4 (27.3; 29.5)^def^	0.9 (0.86; 0.93)^e^	37.2 (35.5; 39)^b^	31.2 (29.9; 32.5)^efg^	1.1 (1; 1.2)^cd^	1.0 (0.9; 1.1)^cd^	2.7 (1.64)
*Mandevilla hirsuta*	20	21.6 (20.2; 23)^def^	20.9 (19.5; 22.3)^ef^	1 (0.92; 1.02)^ef^	17.5 (16.7; 18.3)^def^	17.4 (16.4; 18.3)^ef^	1.0 (0.95 ; 1.04)^de^	18.9 (18; 19.8)^def^	18.5 (17.5; 19.4)^g^	0.7 (0.6; 0.8)^e^	0.7 (0.6; 0.8)^e^	1.7 (0.31)
*Mandevilla rugellosa*	19	10.5 (9.6; 11.4)^g^	14.6 (13.3; 16)^f^	1.4 (1.3; 1.6)^ef^	8.2 (7.3; 9)^g^	10.0 (9.5; 10.5)^f^	1.4 (1; 1.8)^cde^	9 (8.1; 9.8)^f^	11.9 (11.1; 12.8)^g^	0.6 (0.5; 0.7^)e^	0.7 (0.6; 0.8)^e^	0.9 (0.23)
*Petrea volubilis*	30	28.2 (24.6; 31.9)^de^	52.3 (42.7; 61.8)^cde^	2 (1.6; 2.5)^def^	22.7 (21.5; 24)^de^	33.7 (30.4; 37.1)^cde^	1.5 (1.3; 1.7)^cde^	26.2 (23.8; 28.5)^cd^	48.6 (41.6; 55.6)^def^	1 (0.9; 1.1)^bc^	1.3 (1.2; 1.5)^bc^	1.9 (0.62)
*Scleria secans*	23	31.1 (28.7; 33.5)^cd^	173.5 (148; 199)^a^	5.7 (4.8; 6.7)^a^	31.5 (29.5; 33.5)^cd^	105.8 (92; 119.6)^a^	3.4 (2.9; 3.9)^a^	34.4 (32.1; 36.7)^bc^	163.6 (139.7; 187.4)^a^	1.1 (1; 1.2)^ab^	1.5 (1.4; 1.6)^ab^	3.6 (0.34)

The values presented are the averages and their 95% confidence intervals (in brackets) of both directions at the species level. The anisotropy coefficient (basal/apical) for the static force and the sliding force is presented. The table also presents the number of tests performed (N) as well as the average stem diameter per species and its SD (in parenthesis). Different letters indicate significant differences between species.

During a 15 mm slide, the overall mean sliding force is lower than the static force. Despite this, most species manage to deploy at least 10 peaks during sliding that are equivalent to the static force and therefore more likely to check a downward motion during sliding in the basal direction. The stem might slip a little but might find an anchorage point during the slide.

Downward sliding forces were generally lower than downward static forces. Despite this, 6 out of the 10 species developed peaks during sliding that would assure attachment at their maximum reach (Table 2). Four species showed weaker frictional forces during fast movement (*D. kunthii*, *M. hirsuta*, *M. rugosa*, and *I. centricifolius*). This suggests that for these species, the attachment forces are weaker under motion during the entire sliding period than the force required to initiate motion.

The static and sliding attachments on a roughened card substrate represent a relatively conservative substrate with limited anchorage points for microspines to attach via friction or dry adhesion (Gorb, 2008). The substrate probably limited the interactions of the microhook tips with the small asperities offered by the fibres within the card. Further analyses and comparisons should explore how different microhooks would behave on a wider range of substrates. These should include diverse natural structures and surfaces such as twig surfaces, leaf surfaces, and bark. There likely exists a vast range of potential anchoring and interlocking interactions in natural habitats, which would include not only different geometries, such as rounded twigs versus flat leaves, but also different surface properties, such as soft or hard substrates that microspines can penetrate. 

Finally, there was a tendency for the species showing higher static and sliding forces to also show higher numbers of peaks per millimetre during sliding than the species exerting weaker sliding forces (Figs 7, 11).

### Stem ratcheting mechanisms

The anisotropy between apical and basal attachment is believed to be potentially well adapted for ratcheting mechanisms in addition to simply limiting downwards slipping in twining stems (Silk and Holbrook 2005) in the hook-climbing *G. aparine* (Gallenmüller *et al.*, 2009; Bauer *et al.*, 2011) and in the spiny climbing organs of climbing palms (Putz, 1990; Isnard and Rowe, 2008a). The difference between apical and basal sliding and ‘stick’ and ‘slip’ forces possibly even promote ratchet-like attachment during growth and possibly ‘upwards’ net movement of climbing stems in environments where three-dimensional networks of tree branches, shoots, and leaves oscillate in the wind. Certainly, observations show evidence of rubbing and chafing of microspines on branch supports (Supplementary Fig. S13), indicating that canopy movement does displace attached climbing stems bearing microspines.

### Stem shape and microhook deployment

Searcher stems differed in their cross-sectional geometry. Most were circular to elliptical in cross section. *Davilla kunthii* developed flattened, ribbon-shaped searchers with lax or weakly twining climbing behaviour (Figs 1A, 2A; Supplementary Fig. S7B). A departure from a circular cross-section was also noticeable in *S. secans*, which produces triangular cross sections typical of the monocotyledon group Cyperaceae. Both the ribbon-like and triangular stems also produced the strongest static and sliding forces. The geometry of microspine deployment along the extended edges of such stems might well provide concentrated angles of deployment for non-twining and weakly twining climbing behaviour. Microspine deployment might be optimized on stems that have acute edges rather than on rounded profiles.

In climbing palms, circular or adaxial–abaxial patterns appear to be adapted to optimize hook deployment (Isnard and Rowe, 2008a). Stems of *G. aparine* are square-shaped in cross section and have microspines deployed along the edges, and this is also consistent with an ‘edge deployment strategy’ (Bauer *et al.*, 2011). Interestingly, the pattern of recurved spines along the stem ridges of the climbing cactus *Selenicereus setaceus* (Soffiatti and Rowe, 2020) is similar to that seen in *S. secans*, potentially optimizing the deployment of hooks along stem edges but at a much larger scale.

We observed very different patterns of microspine patterning on stems with circular, elliptical, and triangular cross sections. Patterns of microspine deployment in diagonal lines relative to the long axis of the stem potentially provide an improved angle of deployment for attaching to cylindrical supports when in a twined position. We carried out our experiments on flat standard surfaces for comparing multiple species. Future studies could investigate the performance of different microspine patterns and deployment on different geometrical structures relevant to supports in their natural habitats, including trunks, branches and twigs, and leaves.

### Microspine diversity and technical applications

This study was intended to examine the diversity of microspine deployment and its potential ecological significance, but we were also motivated to understand how microspine functionality might be transferred to new technical applications based on climbing plants. There is much interest in microspine attachment properties for new technical applications (Asbeck *et al.*, 2006; Burris *et al.*, 2018; Fiorello *et al*., 2018, 2020a, 2020b). In soft robotics applications, attachment of a climbing or roving artefact to its substrate is necessary to provide an initial anchorage as a bracing mechanism prior to the deployment of more permanent attachment mechanisms.

Bringing biomechanics into the field is especially illuminating for understanding functional attributes such as microspine attachment and how it relates to actual growth habits, in this case, climbing strategies in natural environments. The approach uncovers real-world, sometimes chaotic and unpredictable environmental conditions that climbing plants must deal with. This is particularly the case in cluttered, geometrically haphazard, moving, oscillating conditions while the plant and its substrates are being blown by wind and pounded by heavy rain.

A field approach has allowed us to discover diverse climbing mechanisms that would probably not have emerged from laboratory experiments on model plant species. Further in-depth studies of measurements made on single hooks, including shear anchoring tests (Chen *et al.*, 2013) and pull-off tests, as well as tests on different natural surfaces, such as stem surfaces and leaves, will provide further information on how different species might be adapted for optimally attaching to specific surfaces (Gorb *et al.*, 2002).

Our study pinpointed a number of following potential innovations. First, ‘perfect’ twining behaviour for a potential vine-like artefact might at first sight seem to be the archetypal way for climbing plants to climb through complex environments. Many laboratory experiments have studied helical twining behaviour, the forces operating, and the evident practicality of twining by gripping around a symmetrical surface (Silk and Holbrook, 2005; Isnard *et al.*, 2009). However, in the real world of highly complex, cluttered, unpredictable branching, compounded by interaction with neighbouring stems and leaves, as well as the nearly constant physical movement of these systems, perfect twining is rather rare—at least for any considerable length. Instead, we see that many plants that we would generally refer to as ‘twiners’ actually perform variable kinds of winding, wrapping, and lax twining around a far wider range of supports than symmetrical, cylinder-like, ‘perfect’ supports. So, a strictly ‘twining’ design specification for a technical artefact for climbing and roving through extremely unstructured environments might not be suitable. Perhaps better, as we see in the natural world, would be a more general kind of lax twining, winding, or wrapping mechanism.

Second, our study suggests that microspine deployment facilitates lax twining and scrambling behaviours that are adapted to highly unstructured environments. Non-perfect twiners and scramblers deployed stronger microspine attachments compared with close twining species, which actually deployed much weaker microspine attachments. Microspine deployment is a good attachment device for highly irregular supports.

Third, for both slow movements such as growth and for faster movements during periods of environmental perturbation, high anisotropies between forwards and backwards attachment are highly adapted to facilitate forwards movement and upwards climbing, without slipping downwards. As in previous studies on *G. aparine*, angled microhooks can act not just as very efficient anchorage points but also as very efficient ‘skate-like’ structures for easily sliding in the opposite direction (Bauer *et al.*, 2011; Andrews and Badyal, 2014). Ease of forwards movement is necessary for unencumbered forwards and upwards movement, but at the same time these microhooks act as safety catches in the event of accidental slipping in the wrong direction. These features are of interest for designing low-force, efficient, directional attachment in growing artefacts such as vine-like GrowBots.

Finally, a survey of the diversity of attachment structures such as those of the species covered in our study might have the potential to uncover new and ‘unthought of’ kinds of microspine deployment for technological innovations. One of the species included in our study, *I. centricifolius*, showed an entirely different kind of climbing habit, producing spines that point ‘the wrong way’, towards the apex. Although this form of spine deployment is not fully understood, it underlines the principle that such functional outliers may provide completely novel design ideas for entirely new technical applications—in this case not for a design that ‘grows’ upwards but perhaps for something that stabilizes exploratory shoots that need to climb down. This study focused on the diversity of microspine features and their types of deployment among 10 different species in the same geographical setting of a lowland rainforest in French Guiana. The sampling represents only selected species and not by any means the entire diversity of such systems in this area or in other ecological settings. We hope that the approaches and ideas we have developed might stimulate further studies from both biological and technical points of view in order to more fully understand the true diversity of such systems and their potential transfer towards new kinds of technical application.

## Supplementary data

The following supplementary data are available at *JXB* online.

Fig. S1. Selection of climbing plant species with searcher shoots bearing microspines.

Fig. S2. Detail of the close climbing species *Mandevilla rugosa*.

Fig. S3. Detail of the close climbing species *Mandevilla hirsuta*.

Fig. S4. Detail of the lax climbing species *Doliocarpus dentatus*.

Fig. S5. Detail of the lax climbing species *Davilla kunthii*.

Fig. S6. Microspines act as a locking mechanism for upward climbing and horizontal roving.

Fig. S7. Searcher stems of close twining *Byttneria cordifolia* and angular ribbon-like stems of the lax twiner *Davilla kunthii*.

Fig. S8. Typical species force–distance curve obtained during friction tests in the apical direction.

Fig. S9. Microscopic observation of the stem surface and microspines of all the species sampled.

Fig. S10. Detail of an obstacle that has prevented the close twining of *Davilla rugosa* on a vertical branch.

Fig. S11. Stem of *Doliocarpus dentatus* twining up a young treelet of *Cecropia* sp.

Fig. S12. View of a forest margin environment typical of lowland tropical rainforest of French Guiana.

Fig. S13. Rubbing and chafing of host stem by microspines, indicating movement of the climbing stem relative to the host stem.

Table S1. Statistical assessment of differences in parameters between test directions for each species.

Table S2. Morphometric parameters of the microspines.

Video S1. Identification of searcher stems with microspines.

erac205_suppl_supplementary_figures_S1-S13_tables_S1-S2

erac205_suppl_supplementary_video_S1

## Data Availability

Data supporting the findings of this study are available at Zenodo. https://zenodo.org/record/6543291#.YpGAWqjMJPY; Lehnebach *et al.* (2022).
