# Supplementary material for: Microspines in tropical climbing plants: a small-scale fix for life in an obstacle course
Source: J Exp Bot. 2022 May 13;73(16):5650–70. doi: 10.1093/jxb/erac205 (PMC9467647; doi:10.1093/jxb/erac205)

Lehnebach et al., Supplementary Data

Supplementary Figure S1-S13:

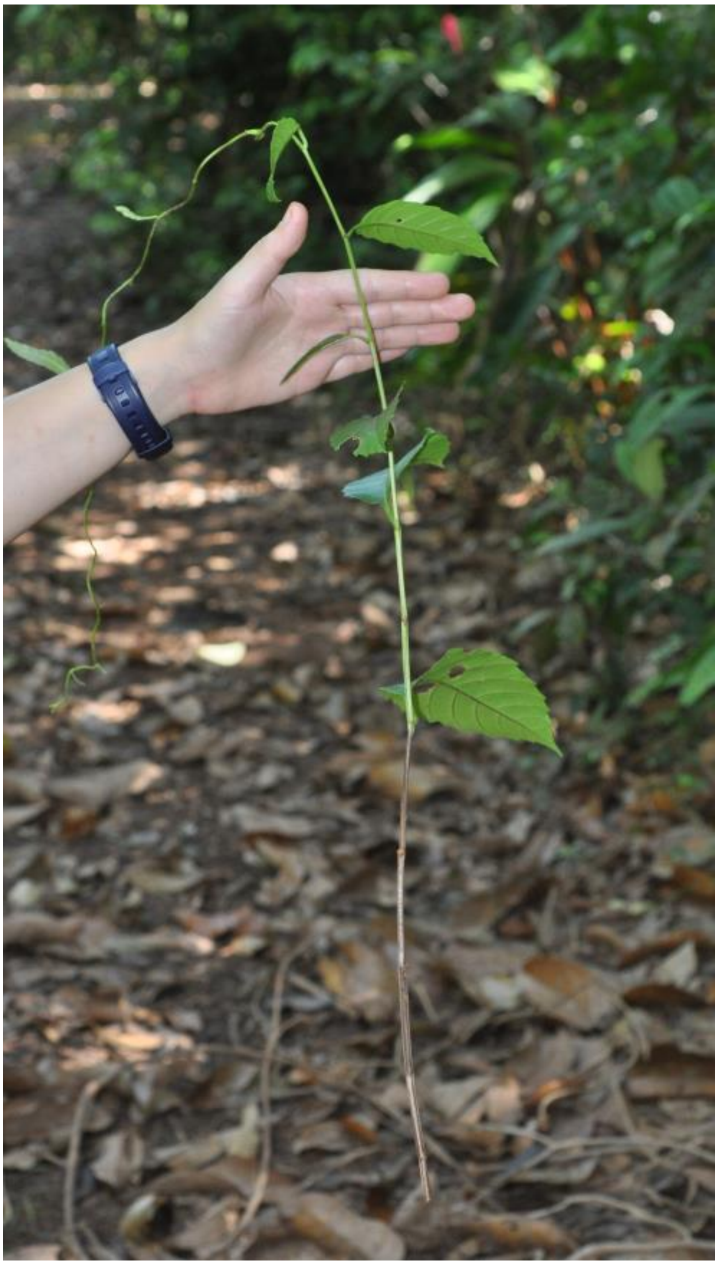

Fig. S1. Selection of climbing plant species with searcher shoots bearing microspines. The stem typically feels sharply and finely textured to the touch especially in a basal direction. Entire searchers can readily attach and fully suspend their entire weight with just a few mm contact with the skin.

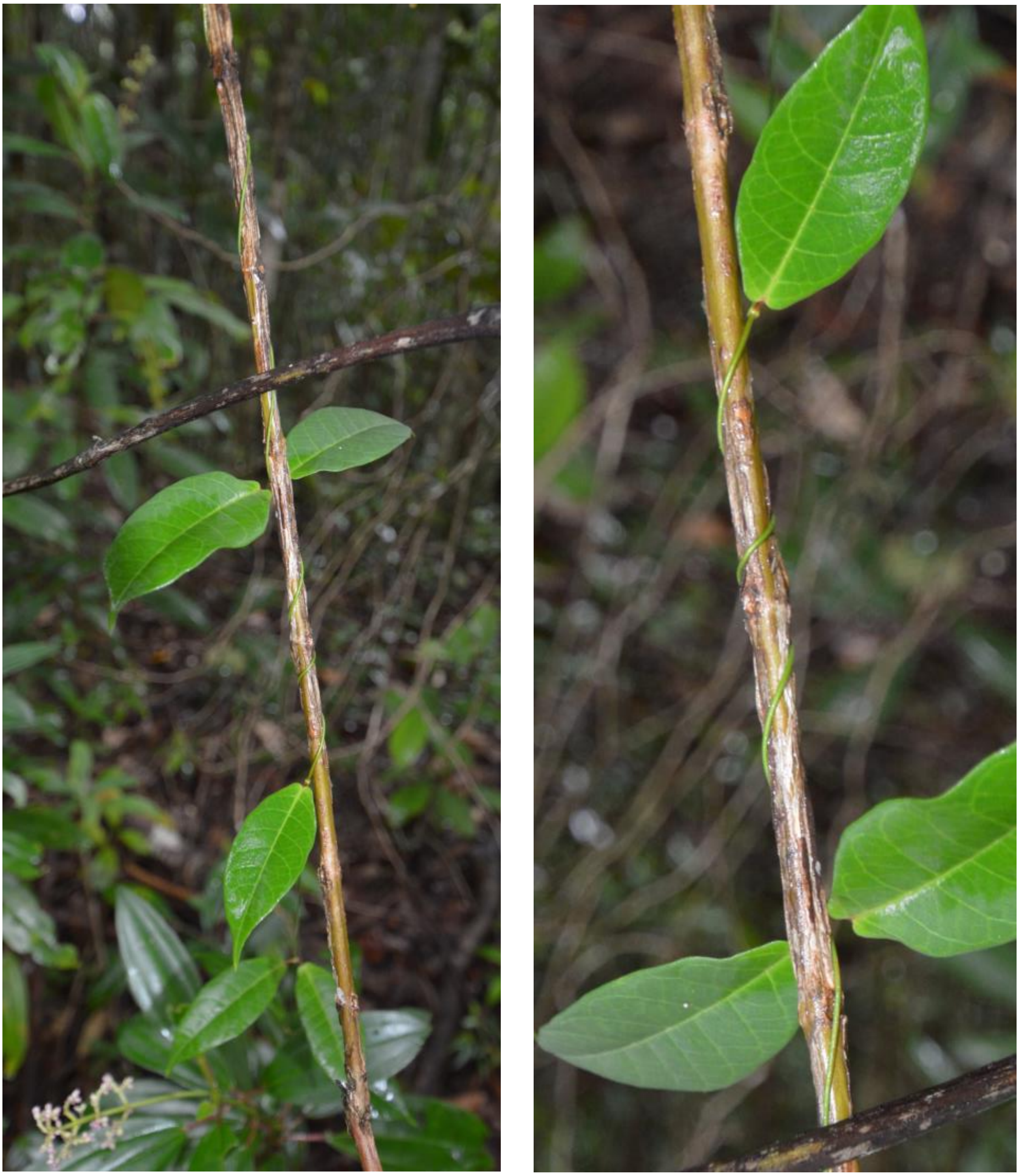

Fig. S2. Detail of close climbing species *Mandevilla rugosa*. The twining is tight against the surface of the host stem for a length of over 40 cm.

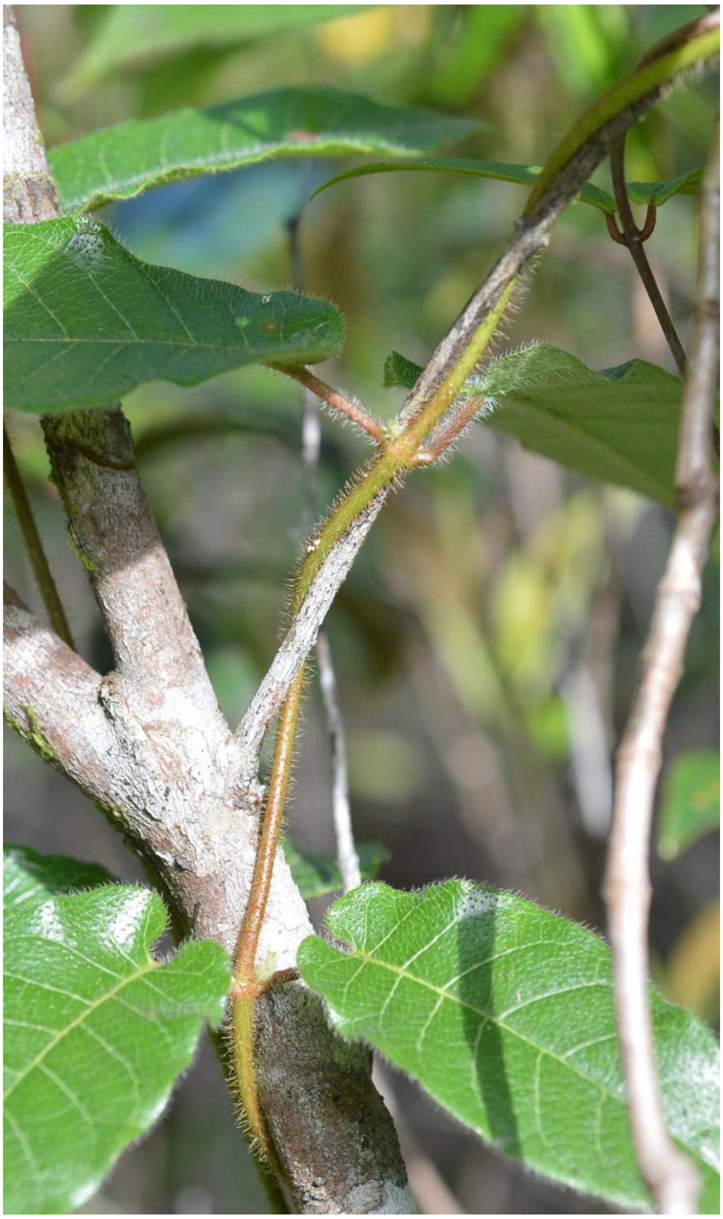

Fig. S3. Detail of close climbing species *Mandevilla hirsuta*. The twining is tight against the lower thicker branch and this earlier twining phase was interrupted by a branch point however the twining continues in close twining on the smaller branch.

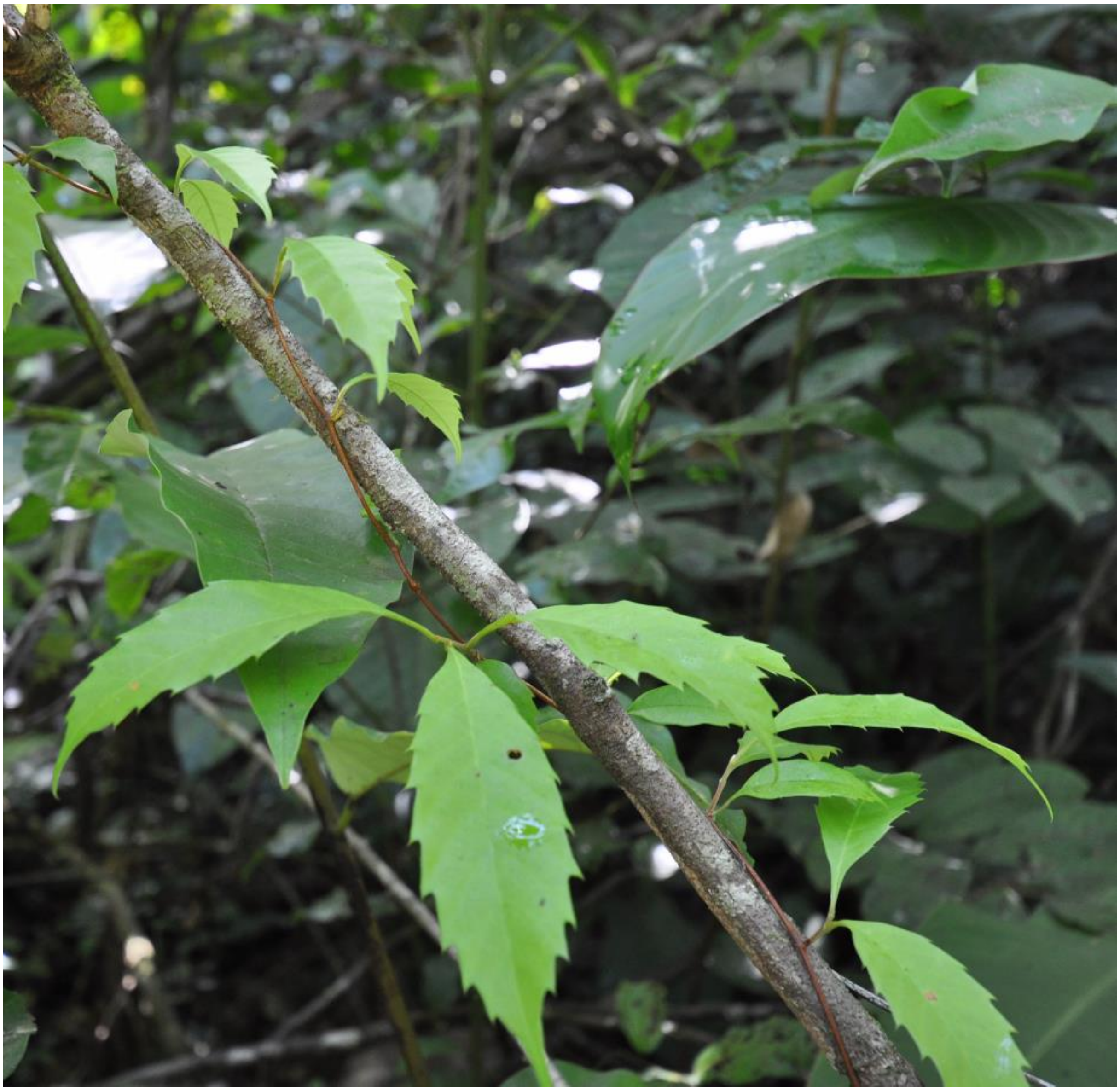

Fig. S4. Detail of lax climbing species *Doliocarpus dentatus*. The twining is extremely loose around the host stem with gaps of up to and over 10 mm. Micro-spines are in contact with the stem support along the stem.

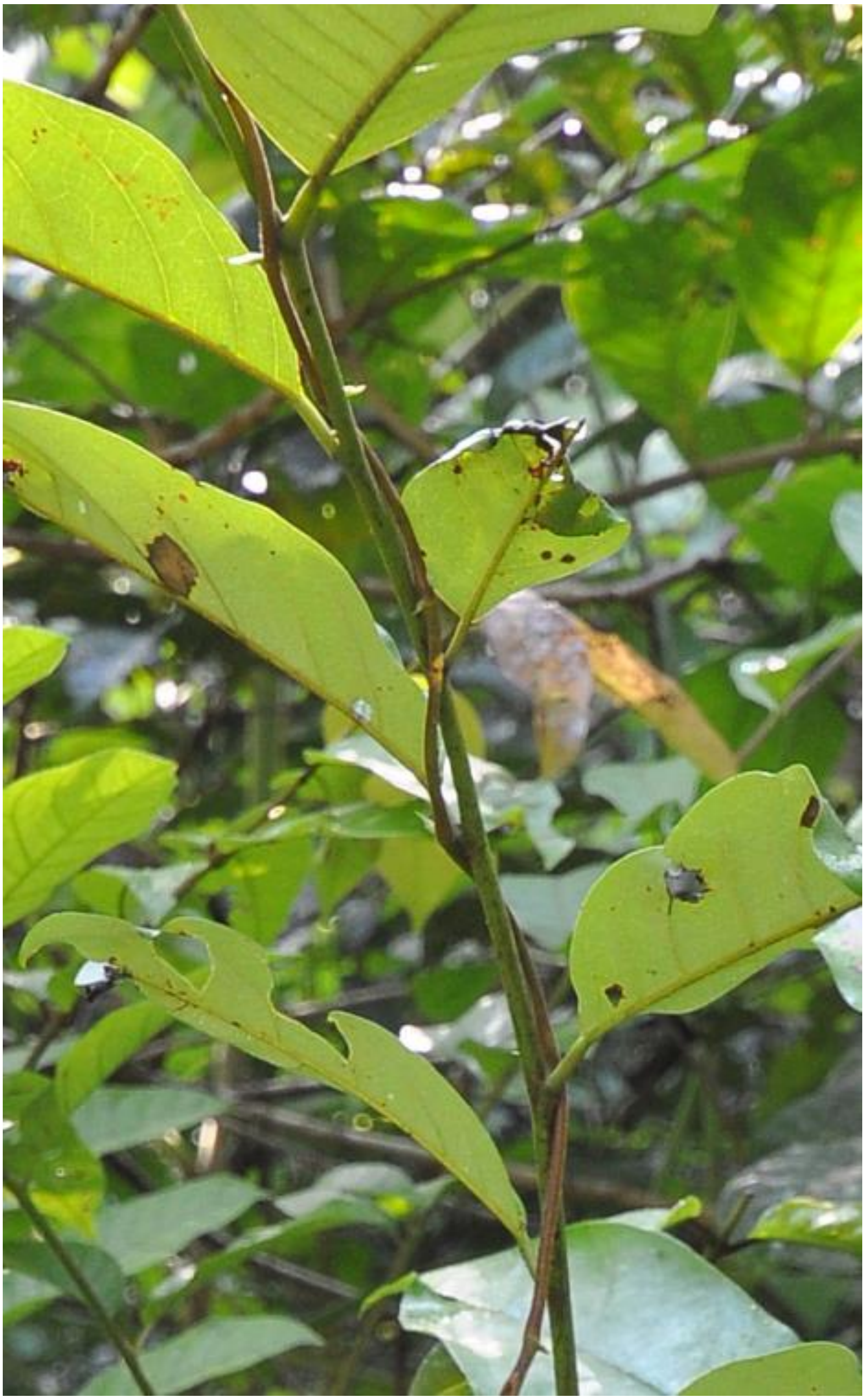

Fig. S5. Detail of lax climbing species *Davilla kunthii*. The twining is relatively loose around the host stem with consistent gaps of 2 to 5 mm. Micro-spines are in contact with the stem support at points of contact.

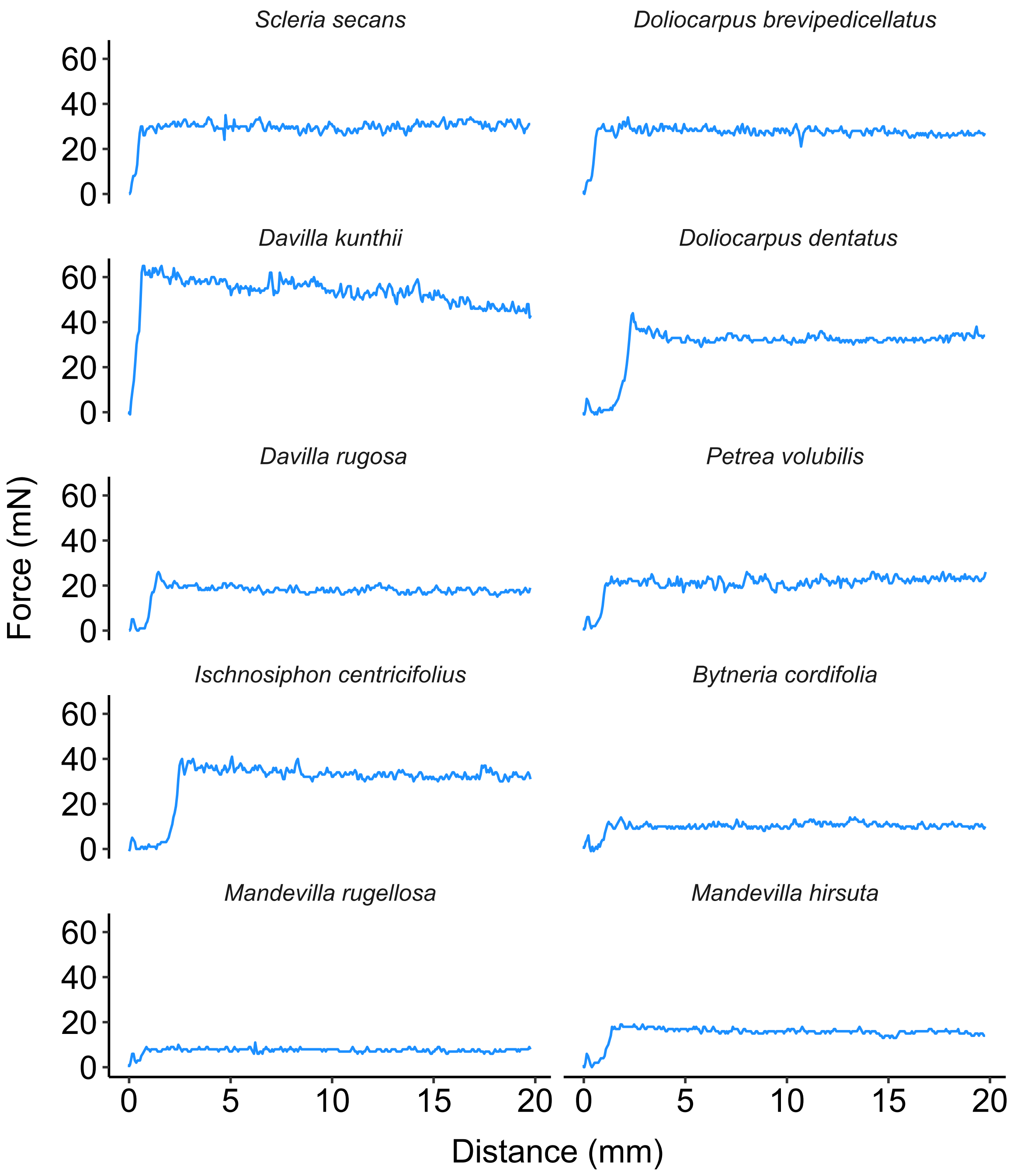

Fig. S6. Typical species force-distance curve obtained during friction tests in the apical direction. Contrary to friction tests performed in the basal direction (see Fig. 6), the variation of the friction force generated during sliding in the apical direction is low and significant peak of forces are not observable suggesting that stick-slip phenomenon in the apical direction does not occur.

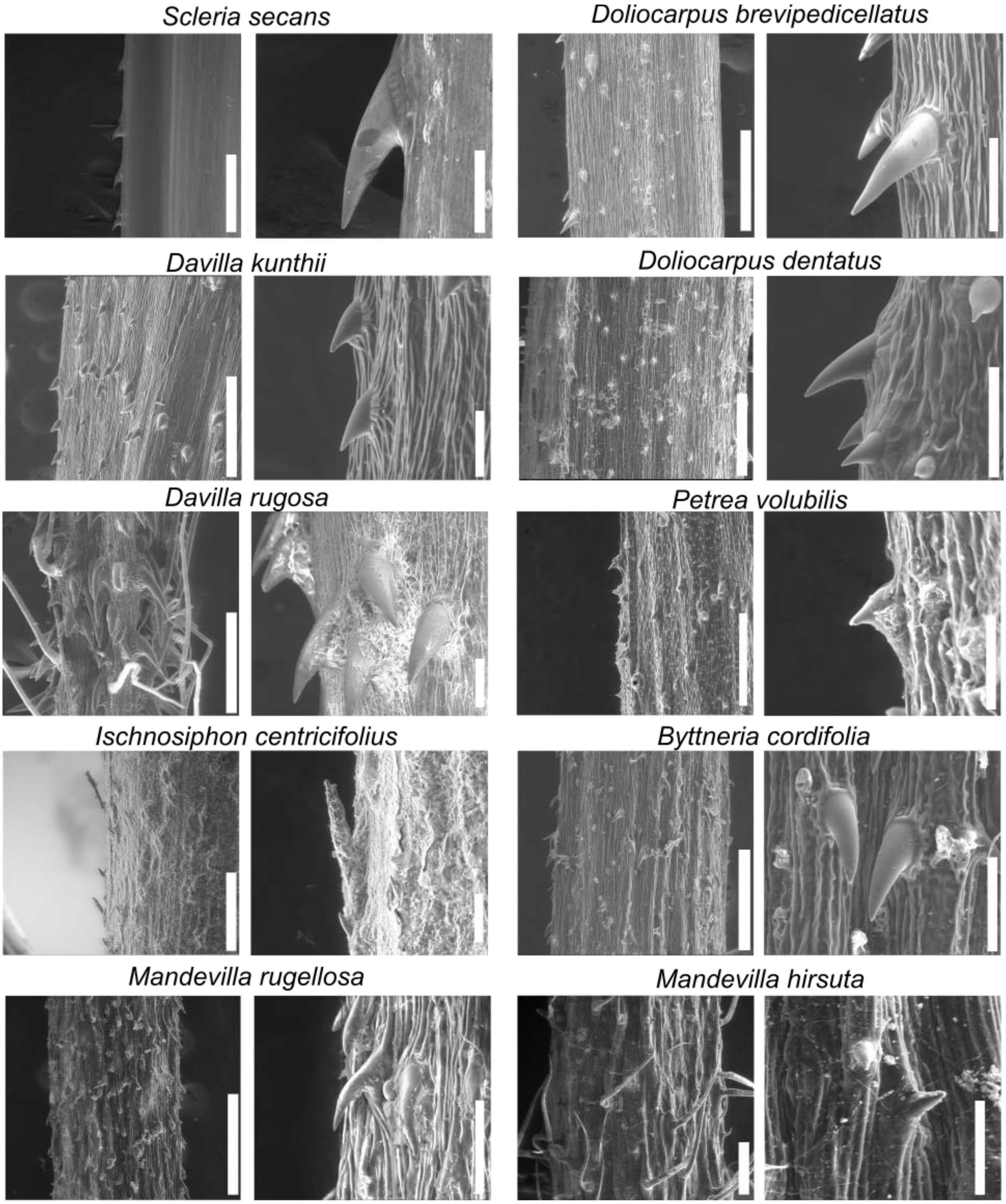

Fig. S7. Microscopic observation of stem surface and microspine of all the species sampled. For each species, SEM micrographs illustrate the surface of the stem (left, scale bar 500 µm) and the morphology of the microspines (right, scale bar 100 µm).

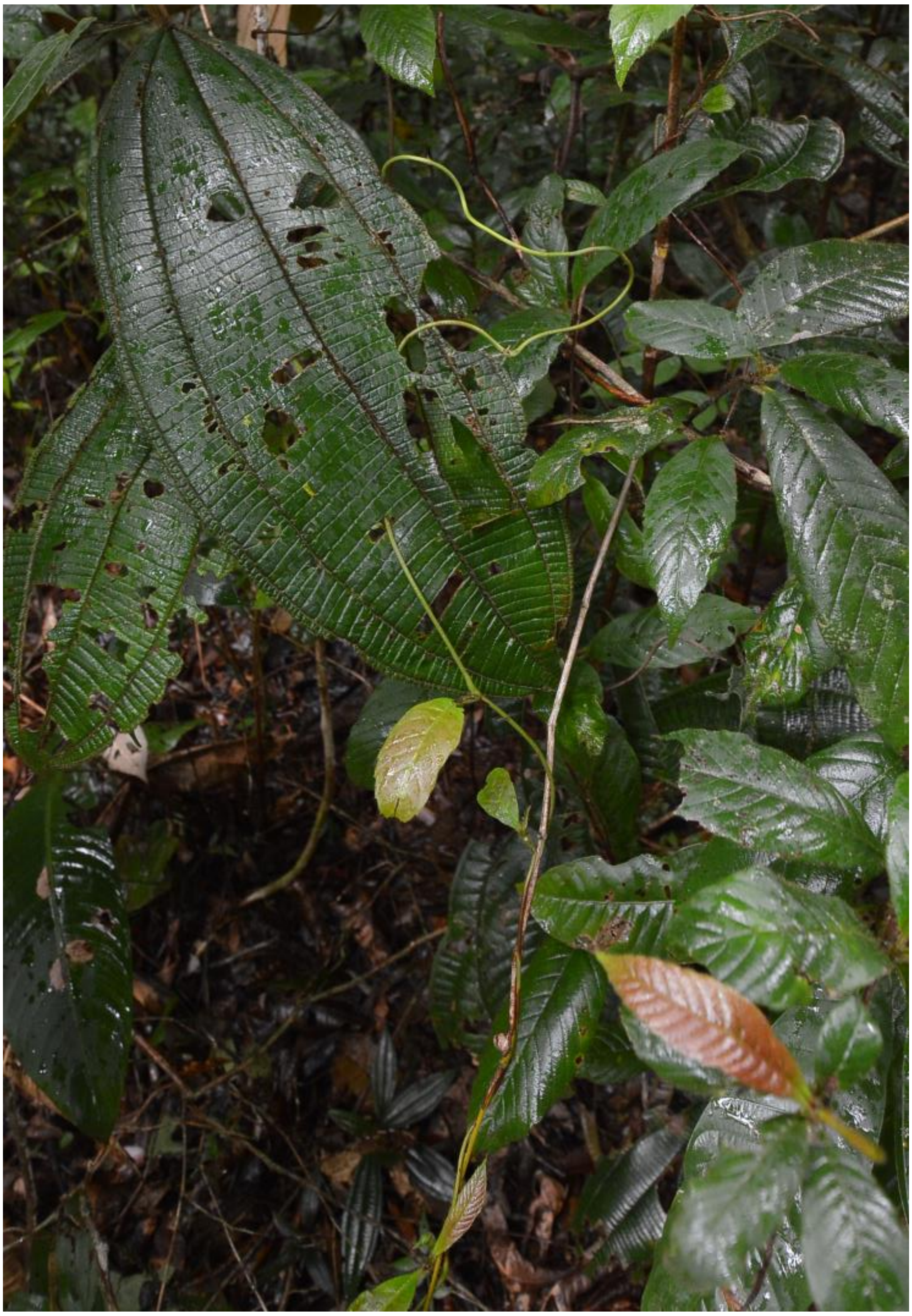

Fig. S8. Detail of an obstacle that has prevented the close twining of *Davilla rugosa* on a vertical branch. A large melastomatacean leaf abuts against the host stem and the searcher has passed through an aperture in the lamina and shows evidence of disorganized twining without finding an appropriate support.

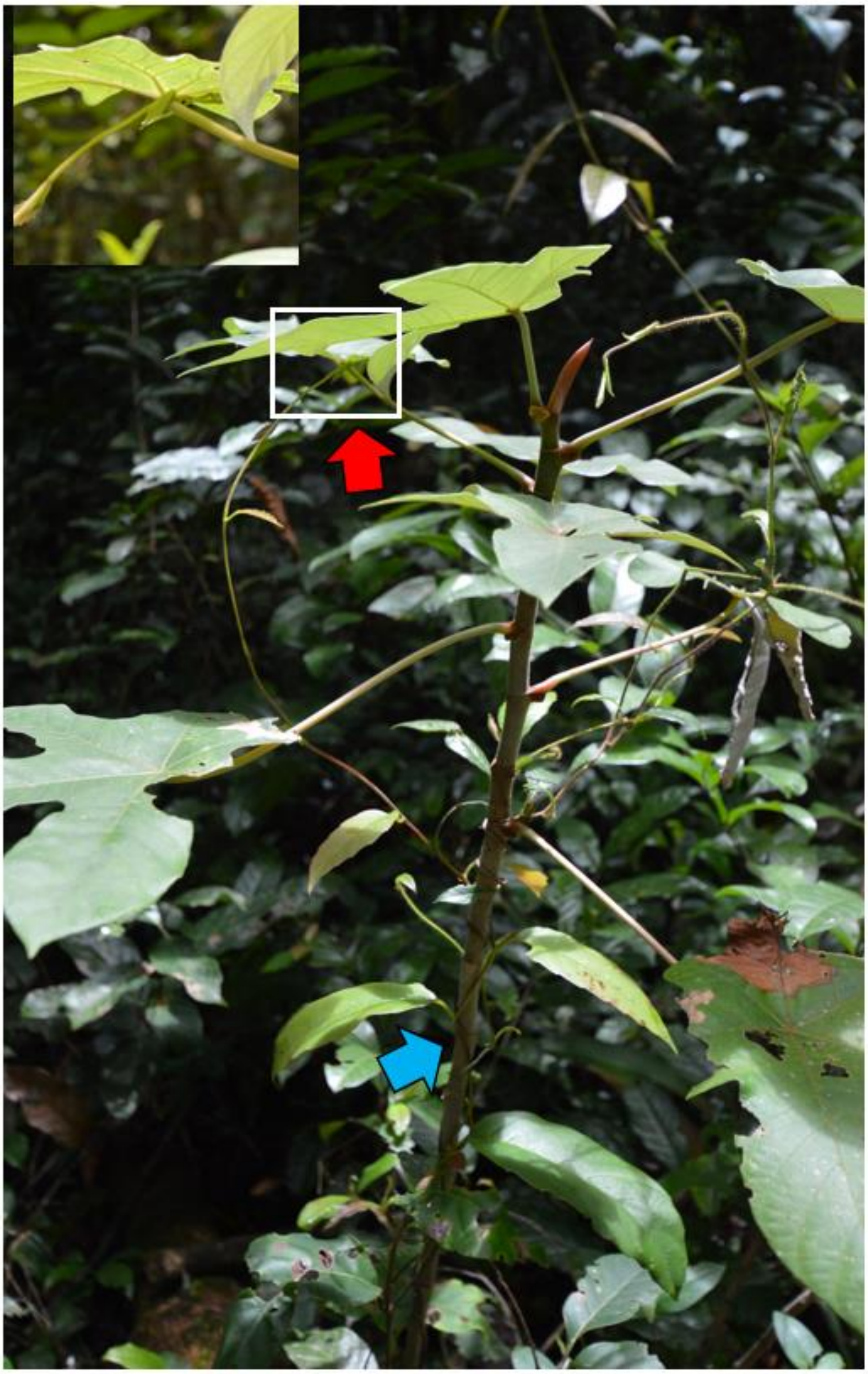

Fig. S9. Twining stem of *Doliocarpus dentatus* twining up (blue arrow) a young treelet of *Cecropia sp.*. The large leaves of the *Cecropia* sp. have deflected the twining stem leader away from the upright stem. This has strongly interrupted the twining behaviour. Near its apex, the searcher has anchored and re-established connection via micro-spine attachment.

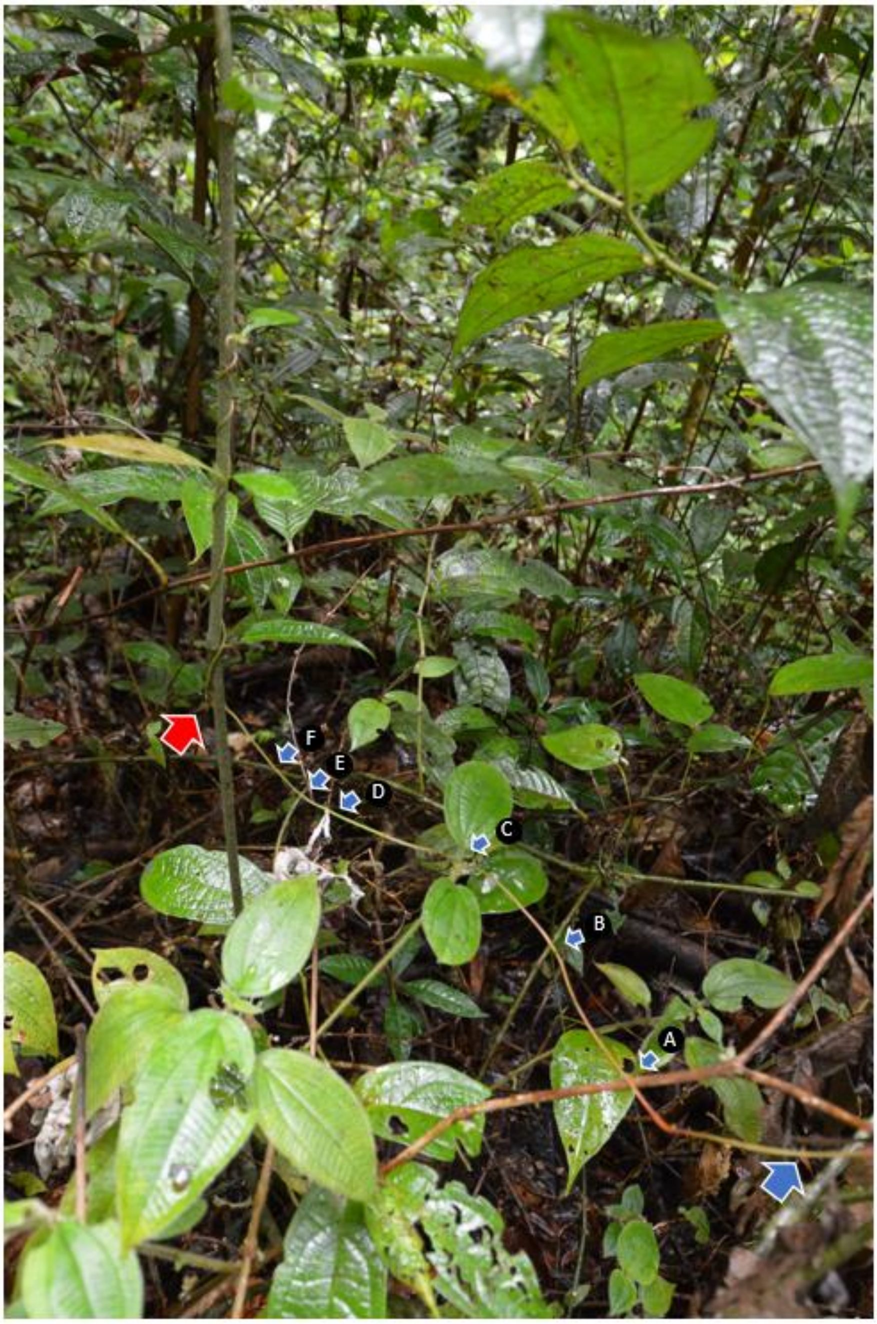

Fig. S10. View of a forest margin environment typical of lowland tropical rain forest of French Guiana. This is a highly unstructured, cluttered and unpredictable three-dimensional organisation. A lax twiner *Doliocarpus dentatus* emerges from the understory at lower right (large blue arrow). It has travelled across six different supports to which it has anchored with microspines and crossed a series of five gaps before attaching and twining onto a slender upright sapling (large red arrow). The supports include Arrow A : a twig with a bark surface. Arrow B: a green twig with a hard smooth axis. Arrow C: a soft apical stem bearing many trichomes of a Melastomataceae shrub; Arrow D: a decayed branch; and Arrows E, F: two further slender twigs with bark surfaces.

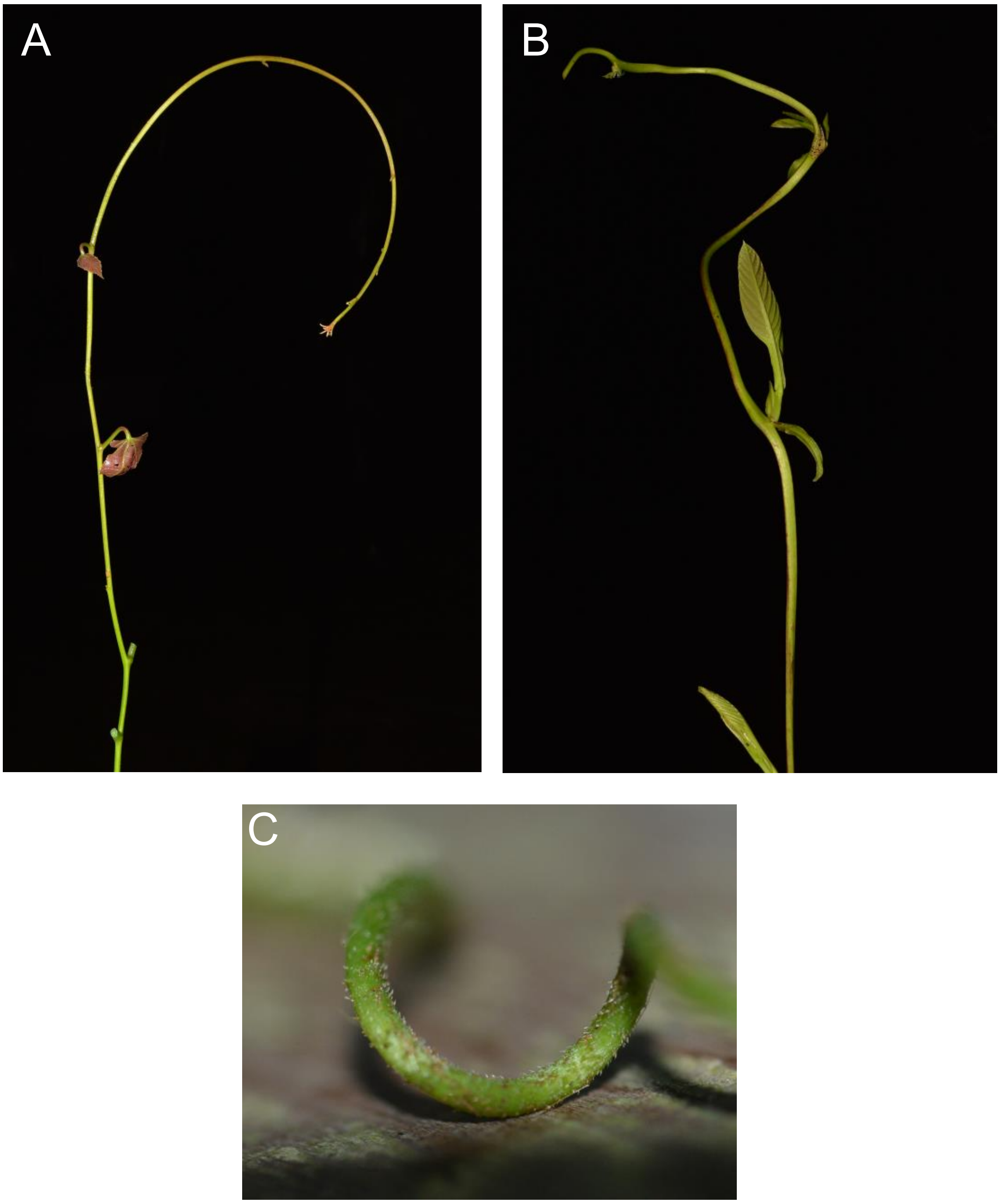

Fig. S11. Searcher stems of (A) close twining *Byttneria cordifolia* and (B) angular ribbon-like stems of the lax twiner *Davilla kunthii*. (C) Recently twined stem segment just below immature green, straight searcher stem of *Davilla rugosa*. The surface of the recently twined stem still retains the textured surface with micro spines typical of the searcher stem before attachment. This indicates that micro-hook attachment is relevant for initial attachment of the searcher and the twining parts of the stem

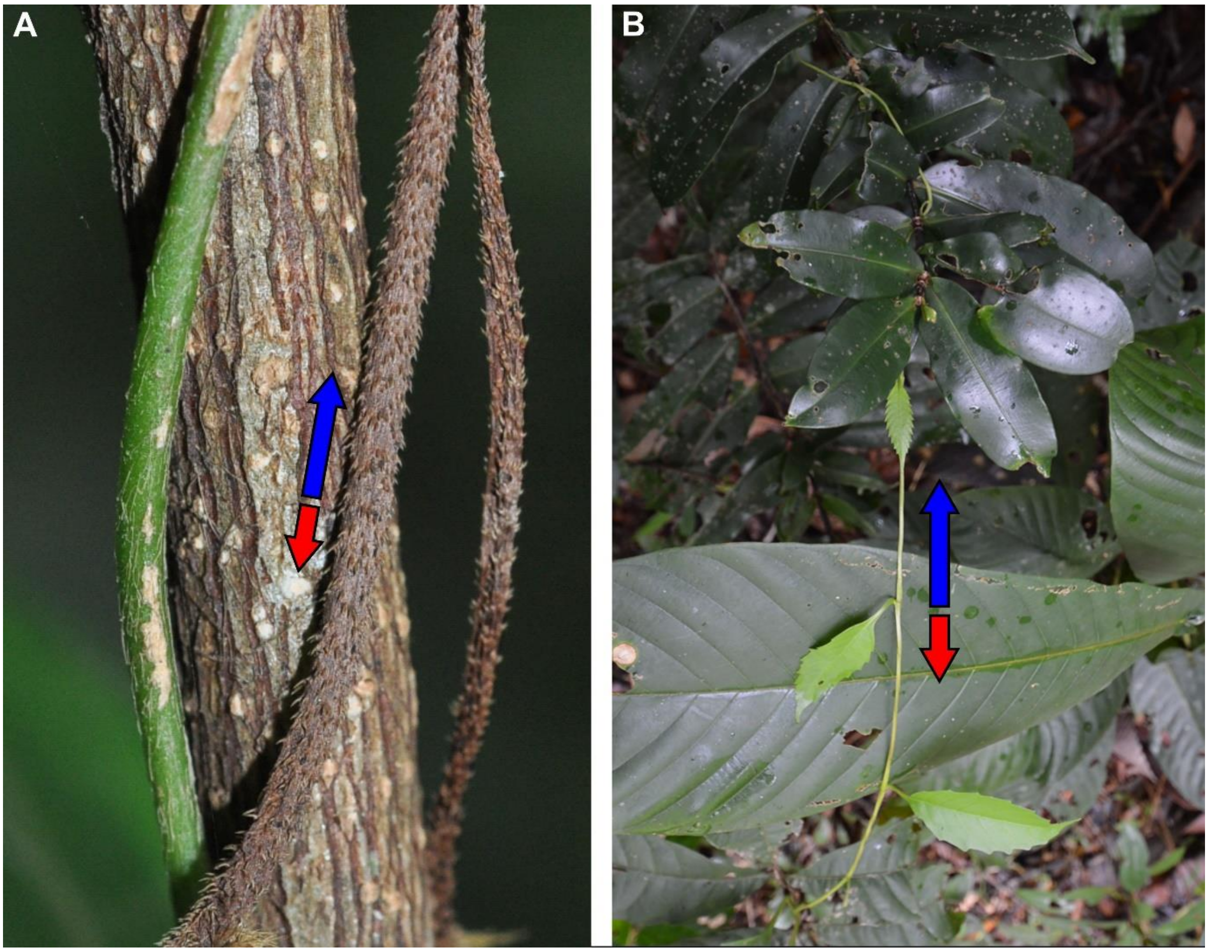

Fig. S12. Microspines act as a locking mechanism for upward climbing and horizontal roving. (A) In upwards climbing movements static friction forces generated by the spines oriented basally will prevent the onset of sliding of the climbing stem and slow-down slipping if sliding starts (red arrow). This is especially true for climbing stems that either do not twine tightly around the support and which perform a more open twining movement. The photograph A shows a liana exhibiting spines that are visible with the naked-eye (i.e. larger than 1 mm). This kind of spines are not considered as microspines (< 1 mm) but are convenient to illustrate the locking mechanism generated by spines whatever their sizes. (B) Apical growth and stem elongation allow the forwards exploration of climbing plant stems across and through highly unstructured environments. Micro-spines oriented basally can permit relatively free movements forwards (blue arrow) while preventing sliding and slipping backward (red arrow). Thanks to the microspines orientation, the liana stem can also take advantages of extrinsic physical movements (e.g. branch or leaf swaying) to initiate a physical ratcheting movement.

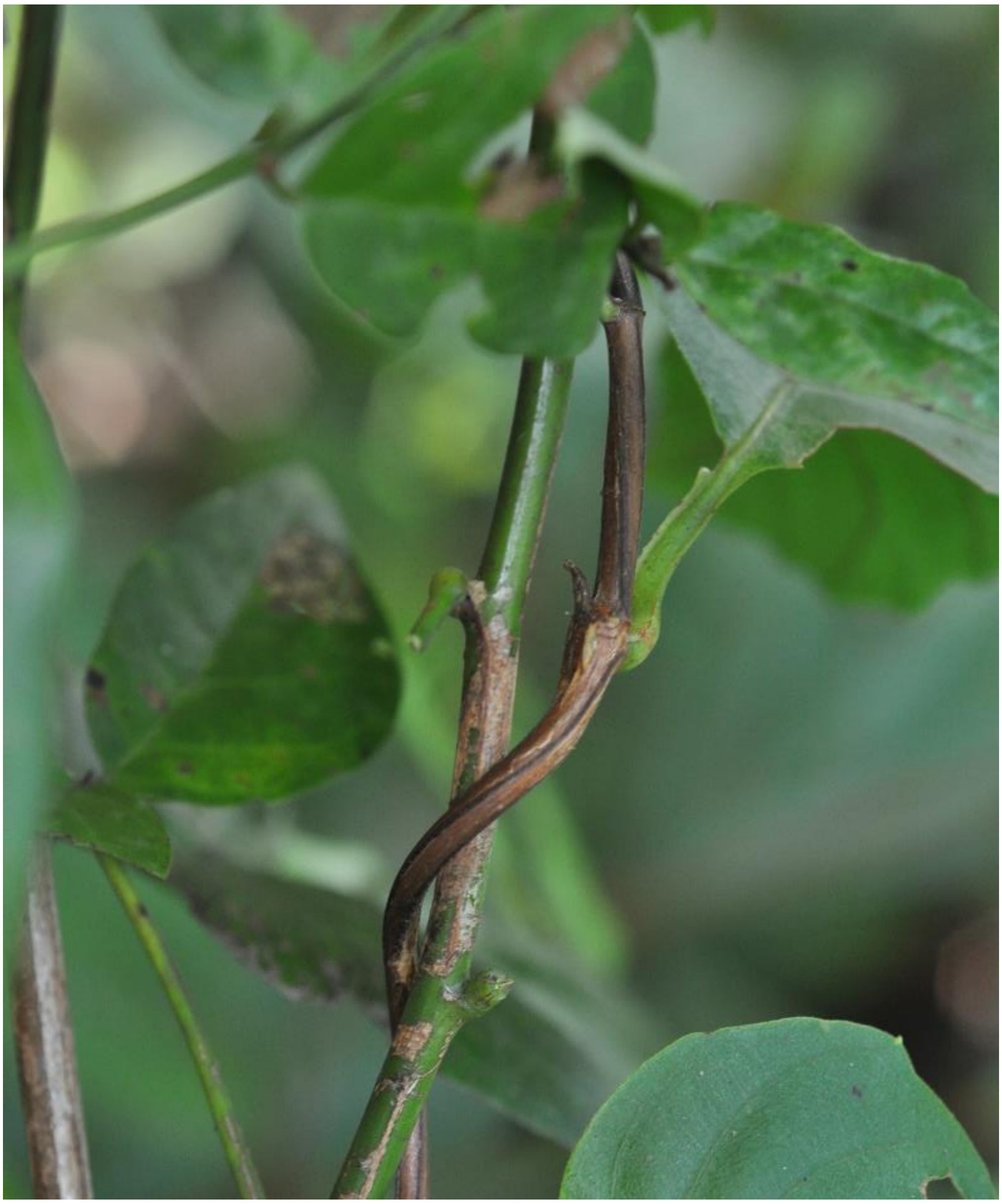

Fig. S13. Lax twining stem bearing microspines loosely twined around host support stem. The host stem shows evidence of rubbing or chafing of the stem likely due to upwards and downwards movement of the climbing plant stem during wind induced movement of small branches in the forest margin environment.

Table S1. Statistical assessment of parameters differences between test directions for each species. The table presents the results of Wilcoxon signed rank sum test performed in the study. For each parameter tested and for each species, the table presents the observed difference and the 95% confidence interval, as well as the test statistic (V), the p-value (P) and the significance (Sig).2

Parameters tested	Species	Diference [CI] (mN)		V	*P*	Sig.
Basal static friction force vs Apical static friction force (Fig. 7A)	*Scleria secans*	142.4	[119;172.5]	276	2.38E-07	*
	Davilla kunthii	109.3	[75;134.5]	405	1.49E-08	*
	Doliocarpus brevipedicellatus	86.9	[58.5;114.5]	274	7.15E-07	*
	Doliocarpus dentatus	45.5	[28;57.5]	325	5.96E-08	*
	Davilla rugosa	38.9	[25;55]	583	1.10E-06	*
	Petrea volubilis	24.0	[11.5;36.5]	388	2.37E-04	*
	Ischnosiphon centricifolius	-6.7	[-10;-4]	64.5	1.98E-04	*
	Byttneria cordifolia	9.1	[6;12]	343	7.45E-07	*
	Mandevilla hirsuta	-0.7	[-2;0.5]	64	3.60E-01	
	Mandevilla rugellosa	4.2	[2.5;6]	181	5.79E-04	*
Basal sliding friction force vs Apical sliding force (Fig. 7B)	*Scleria secans*	74.3	[59.4;88.8]	276	2.38E-07	*
	Davilla kunthii	39.2	[26.4;52.6]	391	1.02E-06	*
	Doliocarpus brevipedicellatus	33.1	[25.1;43.4]	272	1.67E-06	*
	Doliocarpus dentatus	23.8	[16.7;28.9]	325	5.96E-08	*
	Davilla rugosa	14.2	[6.8;17.3]	638	4.32E-08	*
	Petrea volubilis	11.0	[7.3;14.9]	427	1.22E-05	*
	Ischnosiphon centricifolius	-3.7	[-4.9;-2.4]	37	1.33E-06	*
	Byttneria cordifolia	5.2	[3.7;6.6]	347	2.09E-07	*
	Mandevilla hirsuta	-0.1	[-0.8;0.7]	105	1.00E+00	
	Mandevilla rugellosa	1.8	[1;2.3]	189	1.68E-04	*
Basal maximum friction force vs Apical maximum friction force (Fig. 9A)	*Scleria secans*	129.2	[108.2;157.6]	276	2.38E-07	*
	Davilla kunthii	70.2	[49.1;86.6]	406	7.45E-09	*
	Doliocarpus brevipedicellatus	71.2	[55.6;88.7]	275	4.77E-07	*
	Doliocarpus dentatus	40.9	[30.7;46.8]	325	1.31E-05	*
	Davilla rugosa	32.2	[22.5;41.6]	654	2.04E-09	*
	Petrea volubilis	22.5	[14.3;30.5]	433	5.14E-06	*
	Ischnosiphon centricifolius	-6.0	[-8;-4.1]	36	1.30E-05	*
	Byttneria cordifolia	11.2	[8.3;13.6]	349	8.94E-08	*
	Mandevilla hirsuta	-0.4	[-1;0.3]	75.5	2.79E-01	
	Mandevilla rugellosa	3.0	[1.8;3.9]	188	1.14E-05	*
Basal static friction force vs Maximum sliding friction force (Fig. 10)	*Scleria secans*	10.0	[-3.6;22.2]	184	1.70E-01	
	Davilla kunthii	43.4	[28.2;56.9]	389	1.54E-06	*
	Doliocarpus brevipedicellatus	15.2	[-5.4;30.8]	174	2.86E-01	
	Doliocarpus dentatus	7.3	[-4.4;15.8]	204	2.75E-01	
	Davilla rugosa	8.3	[-1.3;14.5]	426	1.48E-01	
	Petrea volubilis	3.6	[-2.3;6.7]	285	2.89E-01	
	Ischnosiphon centricifolius	1.9	[0.2;3.4]	403	2.79E-02	*
	Byttneria cordifolia	-1.0	[-3.4;1.8]	150	5.32E-01	
	Mandevilla hirsuta	2.4	[1.6;3.3]	203	3.62E-05	*
	Mandevilla rugellosa	2.7	[1.4;3.9]	177	3.36E-04	*

Table S2. Morphometric parameters of the microspines. The table presents the average spine density (i.e. the number of spines per mm²), together with the diameter (d), length (l) and tip diameter (td). For each parameters the standard deviation is also presented. The drawing illustrates how diameter, length and tip diameter of the spine were measured. Due to the presence of trichomes, these parameters were not measured for *Mandevilla hirsuta*. Spine-level measurements were performed on 30 spines among 10-12 stems while spine density was measured on 30 stem regions of 0.1 mm² among 10-12 stems.

**Species**	***Spine density (sd) [µm]***	**Diameter (sd) [µm]**	**Length (sd) [µm]**	**Tip diameter (sd) [µm]**
Davilla kunthii	*32.2* (13.1)	53.4 (12.7)	81.2 (16.5)	7.78 (2.73)
Doliocarpus dentatus	71.8 (28.1)	*36.3 (11.5)*	*71.8* (20.4)	4.83 (1.85)
Byttneria cordifolia	27.7 (17.9)	31.6 (11.4)	68.8 (17.7)	5.24 (1.76)
Doliocarpus brevipedicellatus	56.0 (26.5)	37.2 (13.6)	80.5 (23.2)	5.15 (4.33)
Davilla rugosa	33.7 (30.8)	72.7 (10.9)	159.6 (29.6)	6.28 (2.75)
Mandevilla rugellosa	117.7 (38.3)	35.3 (4.7)	56.5 (14.8)	3.51 (1.16)
Mandevilla hirsuta				
Petrea volubilis	34.0 (17.1)	45.2 (17.9)	71.7 (21.6)	6.17 (2.39)
Scleria secans	6.3 (5.7)	60.4 (12.3)	120.9 (39.1)	7.64 (3.56)
Ischnosiphon centricifolius	14.4 (9.3)	33.9 (6.2)	84.0 (21.0)	9.70 (4.27)

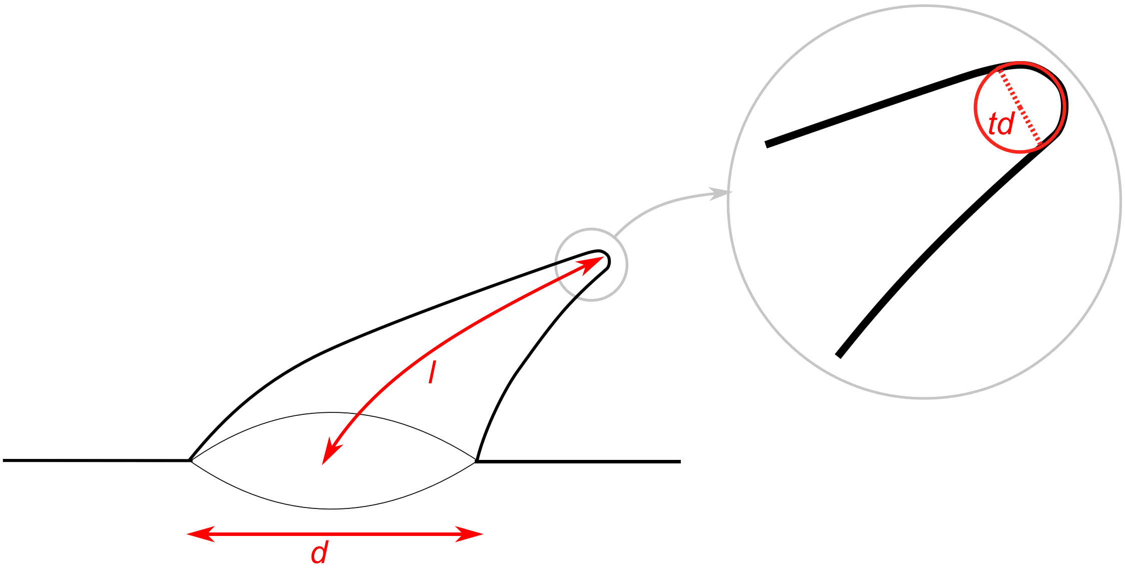

Supplement: erac205_suppl_supplementary_figures_S1-S13_tables_S1-S2 [file erac205_suppl_supplementary_figures_s1-s13_tables_s1-s2.pdf]
